# Effects of the antidepressant medication duloxetine on brain metabolites in persistent depressive disorder: A randomized, controlled trial

**DOI:** 10.1371/journal.pone.0219679

**Published:** 2019-07-19

**Authors:** Ravi Bansal, David J. Hellerstein, Siddhant Sawardekar, Joseph O’Neill, Bradley S. Peterson

**Affiliations:** 1 Institute for the Developing Mind, Children’s Hospital Los Angeles, Los Angeles, CA, United States of America; 2 Department of Pediatrics, Keck School of Medicine at the University of Southern California, Los Angeles, CA, United States of America; 3 Depression Evaluation Service, Division of Clinical Therapeutics, New York State Psychiatric Institute, New York, NY, United States of America; 4 Department of Psychiatry, Columbia University College of Physicians and Surgeons, New York, NY, United States of America; 5 Division of Child and Adolescent Psychiatry, University of California-Los Angeles, Los Angeles, CA, United States of America; 6 Department of Psychiatry, Keck School of Medicine at the University of Southern California, Los Angeles, CA, United States of America; Medical University of Vienna, Austria, AUSTRIA

## Abstract

**Background:**

To assess whether patients with Persistent Depressive Disorder (**PDD**) have abnormal levels of *N*-acetyl-aspartate (**NAA**) and whether those levels normalize following treatment with the antidepressant medication duloxetine. Furthermore, we conducted *post hoc* analyses of other important brain metabolites to understand better the cellular and physiological determinants for changes in NAA levels.

**Methods:**

We acquired proton (1H) magnetic resonance spectroscopic imaging (**MRSI**) data on a 3 Tesla (3T), GE Magnetic Resonance Imaging (**MRI**) scanner in 41 patients (39.9±10.4 years, 22 males) with PDD at two time points: before the start and at the end of a 10-week, placebo-controlled, double-blind, randomized controlled trial (**RCT**) of the antidepressant medication duloxetine. Patients were randomized such that 21 patients received the active medication and 20 patients received placebo during the 10 week period of the trial. In addition, we acquire 1H MRSI data once in 29 healthy controls (37.7±11.2 years, 17 males).

**Findings:**

Patients had significantly higher baseline concentrations of NAA across white matter (**WM**) pathways and subcortical gray matter, and in direct proportion to the severity of depressive symptoms. NAA concentrations declined in duloxetine-treated patients over the duration of the trial in the direction toward healthy values, whereas concentrations increased in placebo-treated patients, deviating even further away from healthy values. Changes in NAA concentration did not mediate medication effects on reducing symptom severity, however; instead, changes in symptom severity partially mediated the effects of medication on NAA concentration, especially in the caudate and putamen.

**Interpretation:**

These findings, taken together, suggest that PDD is not a direct consequence of elevated NAA concentrations, but that a more fundamental pathophysiological process likely causes PDD and determines the severity of its symptoms. The findings also suggest that although duloxetine normalized NAA concentrations in patients, it did so by modulating the severity of depressive symptoms. Medication presumably reduced depressive symptoms through other, as yet unidentified, brain processes.

**Trial registration:**

ClinicalTrials.gov NCT00360724.

## Introduction

Persistent Depressive Disorder (**PDD**, termed Dysthymic Disorder prior to Diagnostic and Statistical Manual 5 (**DSM5**), is a chronic, debilitating depressive illness characterized by depressed mood for 2 or more years.[1] The symptoms of PDD lie along a continuum with those of Major Depressive Disorder (**MDD**) but generally are chronic and milder. Despite its relatively mild presentation, PDD is associated with high levels of functional impairment, disability, and health care utilization.[2] The pathophysiology of PDD is poorly understood and has been inferred largely from studies of MDD, as their treatments are similar and respond to the same medications.[3]

Proton Magnetic Resonance Spectroscopy (**1H MRS**) quantifies the concentrations of several endogenous neurochemicals, including *N*-acetylaspartate (**NAA**), purported to be a marker for the density of healthy neurons;[4] creatine+phosphocreatine (**Cr**), an index for the compounds active in the homeostatic buffering and shuttling of high-energy phosphates, neuroprotection, and perhaps even neuromodulation;[5–9] choline-containing compounds (**Ch**), thought to be an index of membrane turnover and cellular density;[10] and glutamate+glutamine (**Glx**), respectively the major excitatory neurotransmitter and its primary metabolite.[11] MRS studies of MDD thus far have yielded inconsistent findings, with both higher and lower metabolite levels reported in brain regions that support cognitive and emotional processing.

For example, studies of patients with MDD have reported higher Ch in the basal ganglia (**BG**), left dorsolateral prefrontal cortex (**DLPFC**), and left caudate,[12] and frontal lobes,[13] and higher Cr/Ch ratios in the left BG, right prefrontal cortex, and left orbitofrontal cortex.[14] Studies of NAA have reported patients with MDD have lower NAA in the left medial temporal cortex,[15] left hippocampus,[16] frontal white matter, prefrontal cortex, and white matter of the DLPFC;[17] lower NAA/Cr ratio in left white matter of the prefrontal (WMP) lobe but no differences in the Ch/Cr ratio in WMP or Ch/Cr and NAA/Cr in the ACC and hippocampus.[18] Other studies of gamma-aminobutyric acid (GABA) have reported lower GABA in the anterior cingulate cortex (ACC)[19] and across several regions of the brain;[20, 21] yet, another study reported no significant differences in GABA in the occipital cortex;[22] Finally, several studies including a meta-analysis[23] have reported reduced Glx in the subgenual anterior cingulate cortex (sgACC), the anterior cingulate cortex, medial prefrontal cortex, DLPFC white matter, hippocampus, and amygdala,[24] whereas increased Glx in the left hippocampus.[16] These inconsistent prior findings likely derive from differences and limitations in study design. First, most studies measured metabolite levels using single-voxel MR spectroscopy. Although this technique generates spectra with high signal-to-noise ratio, placing a single MRS voxel accurately in a homologous brain region across participants is challenging because of inter-individual variability in brain size, gyrification patterns, and anatomical landmarks for positioning the voxel being imaged. Second, previous studies have not corrected for differing contributions to signal from various tissue types in each MRS voxel. Third, prior studies have typically employed a cross-sectional, case-control design at a single point in time, which can assess only statistical associations, not test putative causal influences between metabolite concentrations, illness, and treatment.[25] Furthermore, brain disturbances in PDD may differ from those in MDD, and therefore abnormalities in PDD cannot be inferred from abnormalities in MDD. We previously showed, for example, that cortical thickness findings were diametrically opposite in persons at familial high risk for MDD compared with patients who had PDD.[26]

We aimed to study the causal mechanisms whereby antidepressant medication changes brain metabolism and symptom severity, while addressing limitations of previous MRS studies. We yoked MRS to a randomized controlled trial (**RCT**) of medication therapy, the paradigmatic study design in modern medicine that supports the causal inference that an active intervention changes outcome measures relative to a control intervention. The RCT study design also permitted us to assess how changes in metabolite levels related to medication-induced changes in symptom severity. As a further advance, we used proton multi-planar chemical shift imaging (MPCSI) to measure metabolite concentrations across most of the brain, while correcting concentrations in each voxel for the contributions of differing tissue types.

## Materials and methods

### Study design

We conducted a 10-week, prospective, placebo-controlled, double-blind RCT in 41 patients with PDD.[27] Patients were assigned randomly to receive either the serotonin-norepinephrine reuptake inhibitor duloxetine or placebo for 10 weeks during the trial. We acquired MRS scans at baseline and at the end of the trial. In addition, we acquired MRS once in 29 healthy controls who were age- and sex-matched to patients. Our primary outcome measure was change in NAA concentrations, the metabolite with the largest and most accurately measured peak in the proton MR spectrum, and an index of mitochondrial and brain-energetic metabolism[28] as well as the density of viable neurons within each voxel.[29] We previously showed in this same cohort that duloxetine produced significantly greater symptom improvement compared with placebo,[27] normalized baseline hyperconnectivity in the default mode network,[30] and normalized thickening of the cortex observed at baseline.[26] Based on our previous findings,[26, 30] we hypothesized that baseline NAA concentrations would differ in PDD patients relative to healthy controls, and that these levels would normalize in duloxetine-, but not in placebo-, treated patients by the end of the trial. Full trial protocol is available as supporting material. The study was approved on May 25^th^, 2006, by the Institutional Review Board at New York State Institute/Columbia University, New York, NY, and we screened our first patient on July 26^th^, 2006. Congress passed the Food and Drug Administration Modernization Act of 1997 (FDAMA), which required trial registration for patients with serious or immediately life-threatening diseases or conditions (https://clinicaltrials.gov/ct2/about-site/history#CongressPassesLawFDAMA). Persistent depressive disorder is not an immediately life-threatening disease or disorder, and therefore, our study of patients with PDD was not covered under FDAMA. Nevertheless, we submitted the study to Clinicaltrials.gov on August 3^rd^, 2006 and received approval on August 7^th^, 2006, and started the randomized clinical trial on August 17^th^, 2006, after the study was registered with and approved by Clinicaltrials.gov. One year after our trial was already registered, Congress expanded the requirements for registering clinical trials in 2007 by passing Food and Drug Administration Amendments Act of 2007 (FDAAA), which required more types of trials, including ours, to be registered (https://clinicaltrials.gov/ct2/about-site/history#CongressPassesLawFDAAA). The authors confirm that all ongoing and related trials for this drug/interventions are registered.

### Participants

**Fig 1:**
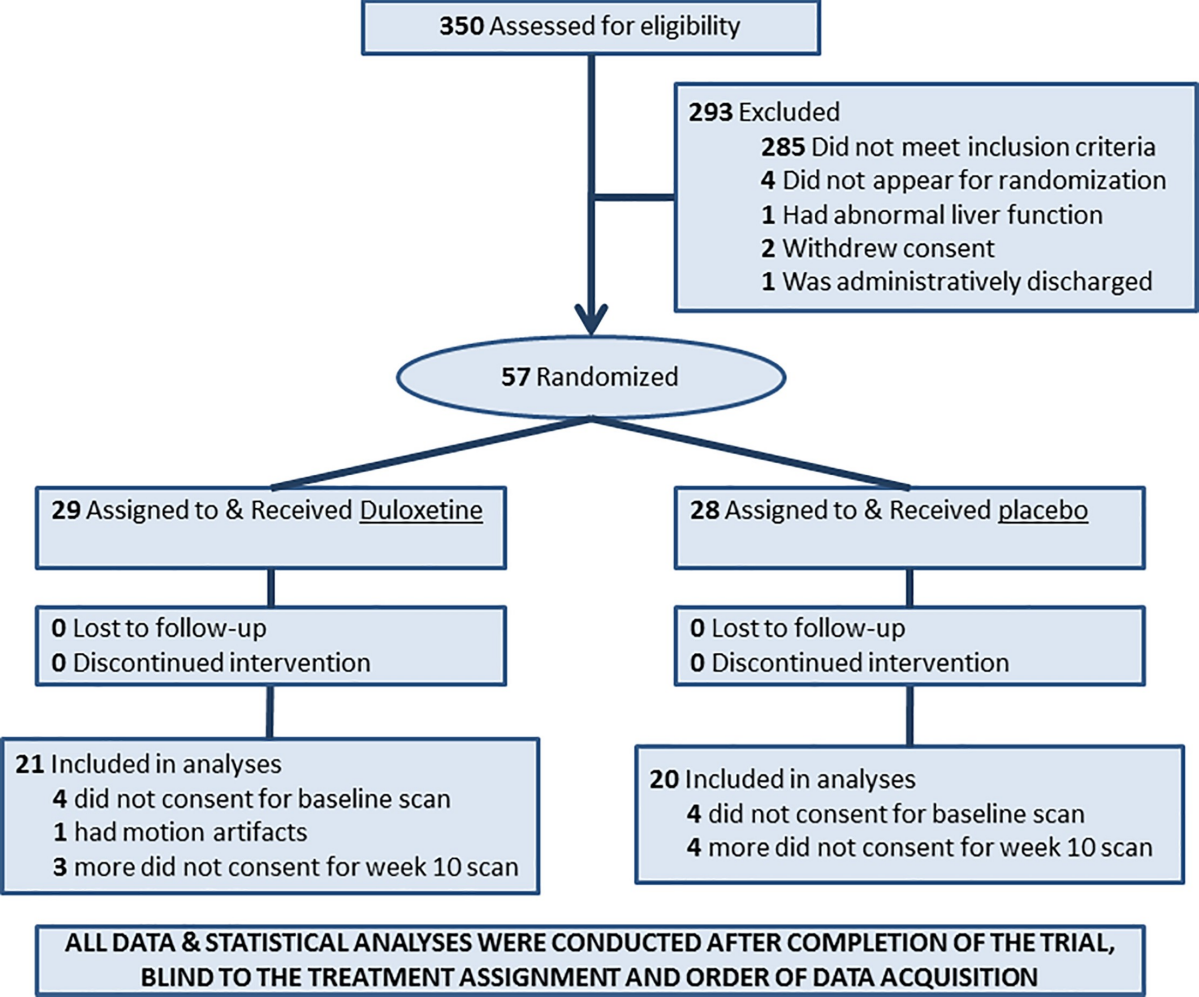
CONSORT Diagram for the Randomized Controlled Trial (RCT). We enrolled 57 patients and acquired MRS data in 41 patients at two time points: at a baseline before the start of the trial and immediately following the end of the trial. All patients using psychotropic medications (N = 4) underwent a 4-week wash out period before starting the trial. In addition, we acquired MRS data at one time point in 29 healthy controls. The patients were randomized to receive either the active medication duloxetine or placebo for 10 weeks. Once the trial was complete and MRS data were acquired in every participant, we processed MRS data blind to the order of data acquisition, participant characteristics, and patient assignment to a treatment arm. Subsequently, all statistical analyses were conducted on the processed MRS data.

Recruitment and assessment procedures, and inclusion/exclusion criteria for patients and healthy controls, are described in detail elsewhere.[27] We started patient assessment and recruitment in July 2006 at the Depression Evaluation Service of the NY State Psychiatric Institute (NYSPI) and ended the follow-up for the last participant in November 2011. Overall, 350 patients expressed interested in the double-blind study, who were assessed by experienced research psychiatrists with more than 20 years of experience in conducting clinical trials for mood disorder. Inclusion criteria for the study included a 24-item Hamilton Depression Rating Scale (**HDRS**)[31] score > = 12 at baseline and the DSM5[1] criteria for PDD. Of the 65 patients who met the eligibility criteria, 57 patients participated in the study. Depressive symptom severity was assessed using both the **HDRS**[31] and the Cornell Dysthymia Rating scale (**CDRS**).[32] Although some patients had HDRS scores higher than 20, they did not meet the DSM criteria for MDD (requiring > = 5 of 9 category A symptoms) and all had depression for at least 2 years. All patients agreed not to take any other psychotropic medications or to receive psychotherapy during the 10-week trial. We acquired MRS data at baseline in 53 patients; however, 11 refused MRS when completing the trial. All participants were adults and remained still during the MRI scan. Therefore, MRS data for only 1 patient had motion artifact. We therefore had useable MRS data at both time points in 41 patients. In addition, we acquired MRS data once in 29 healthy age- and sex-matched controls (**Table 1 & Fig 1**). The healthy controls were randomly selected from a purchased telemarketing list of 10,000 individuals who were of the same age range and lived in the same neighborhood as patients. Individuals who are age- and sex-matched to patients were contacted by sending a letter introducing the study and then following up with a phone call. All participants provided written informed consent.

**Table 1:** Participant demographics and symptom severity. Dysthymic patients did not differ significantly from healthy controls in age (p = 0.41) or sex (*χ*^2^ = 0.17, df = 1, p = 0.68). Duloxetine-treated patients did not differ from placebo-treated patients in age (p = 0.44), symptom severity (p = 0.26), or lifetime history of Major Depressive Disorder (*χ*^2^ = 1.17, df = 1, p = 0.27), but differed significantly on sex (*χ*^2^ = 7.15, df = 1, p = 0.007). The excellent remission rate in duloxetine-treated patients is likely because of their treatment with a high dose (~95 mg/day) of duloxetine. *Responder* is defined as a patient whose symptom severity at week 10 decreased by 50% or more relative to its baseline severity. The table lists the average and standard deviation for age and symptom severity.

		Patients		Healthy (N = 29)
		Duloxetine (N = 21)	Placebo (N = 20)	
**Age (years)**		39.1±10.0	40.8±10.8	37.7±11.2
**Sex**		7 Males	15 Males	17 Males
**Sx, Baseline** **CDRS**				
	**HDRS-24**	20.7±4.1	22.2±4.8	n/a
	**HDRS-17**	14.1±3.8	14.9±35.	n/a
	**CDRS**	37.6±8.2	38.4±8.2	n/a
**Sx, Week 10**				
	**HDRS**	5.8±3.1	15.8±7.7	n/a
	**CDRS**	12.1±7.4	29.0±13.1	n/a
**MDD, Lifetime/Current**		13/0	9/0	n/a
**GAD, Lifetime/Current**		5/0	3/0	n/a
**OCD, Lifetime/Current**		1/2	1/0	n/a
**PTSD, Lifetime/Current**		0/1	1/0	n/a
**Social Phobia, Lifetime/Current**		0/3	1/4	n/a
**Remitted, Week 10**		15	3	n/a
**Responders, Week 10**		18	3	n/a

**HDRS** = Hamilton Depression Rating Scale; **CDRS** = Cornell Dysthymia Rating Scale; **n/a** = not applicable; **MDD** = Major Depressive Disorder; **GAD** = Generalized Anxiety Disorder; **OCD** = Obsessive Compulsive Disorder; **PTSD** = Post Traumatic Stress Disorder; **Sx** = Symptom Severity

#### Inclusion and exclusion criteria

Inclusion criteria for patients were male or female of age 18 to 75 years with baseline HDRS-24 scores ≥ 12, a current DSM-IV-TR diagnosis of dysthymic disorder or depression not otherwise specified, and deemed likely to be compliant with study procedures. Exclusion criteria for patients were DSM-IV diagnosis of Major Depressive Disorder (MDD) in the past 3 years, Bipolar Disorder, Schizophrenia or other psychotic disorder, dementia or other cognitive impairment, drug or alcohol abuse or dependence within the past 6 months, serious risk for suicide during the course of the study, unstable medical conditions, current or planned pregnancy, current eating disorder, and lack of capacity to consent to study participation. Exclusion criteria for healthy controls included history of medical, psychiatric, or Axis 1 disorders. Additional exclusion criteria for both patients and healthy controls included contraindication for MRI scanning (e.g., metal implants, dental braces, pacemakers), any prior neurological disorder including Autism Spectrum Disorder, current or previous substance abuse, any previous seizure, head trauma with loss of consciousness, and a previously diagnosed intellectual disability defined as IQ < 70.

#### Randomization and masking

Patients were randomized to receive either up to 120 mg/day of duloxetine (active medication) or gelatin shell capsules with sugar balls (placebo) in 30 mg capsules. Individual patient dosing was determined by clinical response. The mean dose of duloxetine was 89 mg/day (standard deviation = 28.3 mg/day), and placebo equivalent was 100.7 mg/day (Standard Deviation (**SD**) = 27.3 mg/day). The study was double blinded, with participants, clinicians, and raters were unaware of patient assignments to treatment arms. Randomization was performed by a statistician who otherwise was not involved in conducting the study. The statistician applied an online random number generator to create a list of 0’s (placebo) and 1’s (medication) in blocks of 20 numbers. A staff member prepared unmarked kits for 10 weeks of treatment with either active medication or placebo. On receiving a patient identification number (**ID**), the statistician sequentially assigned the next number on the list of random 0’s and 1’s to the patient ID, and forwarded an appropriate, unmarked kit to the research psychiatrist. Finally, the statistician stored the patient ID and its treatment assignment in a locked cabinet, which was inaccessible to other study investigators.

### Procedures

#### Magnetic Resonance Imaging (MRI) scanning

MRI data were acquired on a 3 Tesla (**T**) GE Signa MRI scanner equipped with an 8-channel head coil. Anatomical MRI scans received a reading by a neuroradiologist to exclude the presence of clinically significant findings.

#### T1-weighted (T1w) anatomical MRIs

Were obtained using a three dimensional (**3D**) fast spoiled gradient recall (**FSPGR**) sequence with in plane Resolution = 0.976x0.976 mm^2^; Slice Thickness = 1.0 mm; Repetition Time = 4.7 ms; Echo Time = 1.3 ms; Inversion Time = 500 ms; Flip Angle = 11°; Matrix = 256x256; Field of View = 25 cm; Phase Field of View = 100%. We acquired two scans each with Number of Excitations (**NEX**) = 1, which were coregistered and averaged offline. We used these anatomical scans to coregister MRS data into a common coordinate space of a template brain and to calculate metabolite concentrations corrected for partial volumes of gray and white matter in each MRS voxel.

#### Localizer for MRS data

We acquired an anatomical localizer scan in the same slice locations as the MPCSI data with the following parameters: In-plane Resolution = 0.9375x0.9375 mm^2^; Slice Thickness = 10.0 mm; Repetition Time = 300 ms; Echo Time = 10 ms; Matrix = 256x256; Field of View = 24 cm; Phase Field of View = 100%; Number of Slices = 6; Slice Spacing = 2.0 mm. We used these localizer images to coregister each participant’s MRS data into the coordinate space of the corresponding T1w anatomical scan.

#### Proton MRS data

**Fig 2:**
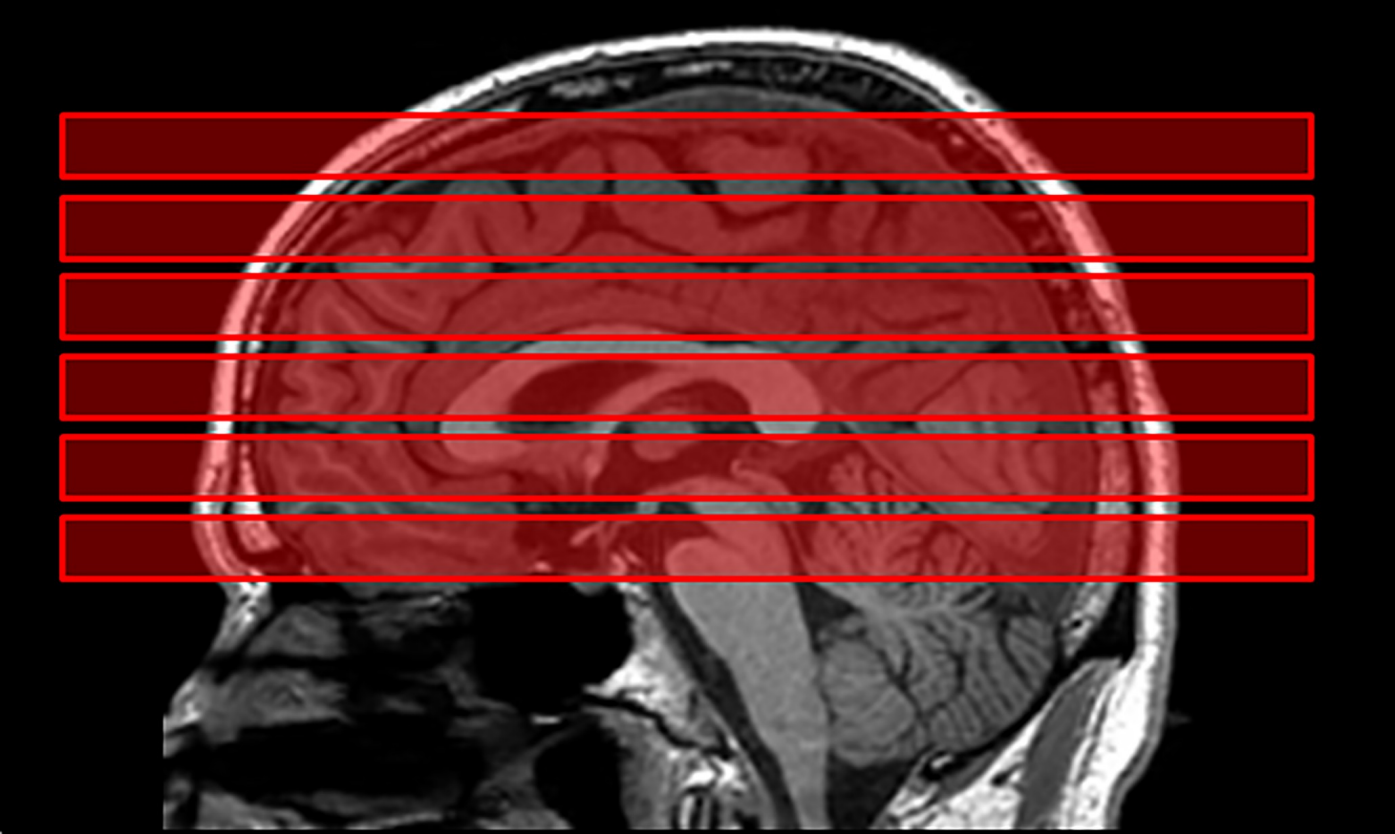
Placement of the Six Slabs in Multiplanar Chemical Shift Imaging (MPCSI). We acquired MR spectroscopy data in six axial oblique slabs through the brain, with each slab comprising a 24 x 24 matrix of 1 cm^3^ voxels with 2 mm gap between adjoining slabs. The second slab from the bottom was placed such that it contained the anterior commissure (AC) and the posterior commissure (PC). We placed one slab below and 4 slabs above the AC-PC slab, thereby generating metabolite maps across the entire brain in each participant.

We acquired the MRS data in 6 axial-oblique slices parallel to the anterior commissure-posterior commissure (**AC-PC**) line, with one slice below, one slice containing, and 4 slices above that line (**Fig 2**). We used an MPCSI^32^ sequence and second-order dynamic shimming with the following parameters: Resolution = 10x10 mm^2^; Slice Thickness = 10.0 mm; Slice Spacing = 2.0 mm; Repetition Time = 2300 ms; Echo Time = 144 ms; Samples = 512 complex points; Number of Phase Encoding Steps = 24x24; Field of View = 24 cm. We suppressed water signal using Chemical Shift Selective (**CHESS**), and we suppressed extracranial lipids using 8 angulated saturation bands around the brain. Scan time was 25 minutes.

#### Processing MRS data

All MRI data were processed blind to the order of scan acquisition (baseline or week 10), treatment assignment, and participant clinical characteristics. The procedures for processing anatomical MRI are described in **S1 File**. We processed MRS data voxel-wise from each of the 8 coils separately before combining the signals to generate spectroscopic images.[33] First, we phase-aligned the signals, smoothed aligned signals using a Hamming window filter, spatially reconstructed the time-domain free induction decay (**FID**) signal in each slice using a 2D Fourier transform, suppressed residual water by applying a singular-value decomposition to the FID signal, applied a 4 Hz Gaussian filter for line broadening, and transferred the time-domain signal into the frequency domain by applying a 1D Fourier transform.[34] Finally, the processed frequency-domain signals from the 8 coils were combined by computing their weighted sum.[33] We loaded the combined signal into the software platform *3DiCSI* (http://hatch.cpmc.columbia.edu/software.html), identified voxels with good MRS signal, and saved their spectral data, eliminating data outside of the brain. We then applied least-squares estimation to fit Voigtian curves to the peaks for NAA, Cr, Ch, and Glx, and estimated metabolite concentrations as the area under the respective fitted curves. We visually assessed spectral quality, rejecting data from voxels showing either strong lipid contamination, insufficient suppression of residual water, unresolved Cr and Ch peaks, or a full width at half maximum >12 Hertz (**Hz**) for any peaks **(S1 Fig)**. We then computed the signal-to-noise ratio (**SNR**) for each metabolite level, estimating background noise as the standard deviation of the real part of the complex spectrum in voxels where metabolite signal was absent. The average SNR for NAA was >280, an excellent value that was attributable to use of the 8-channel head coil. We then generated a spectroscopic image for each metabolite by mapping its ratio of peak area to background noise. This ratio accounted for variations in receiver and transmitter gain across participants.

#### Correction for partial volume effects on metabolite concentrations

We corrected for both the point spread function (**PSF**, the dispersion of MRS signal into neighboring voxels) and partial volume effects (differing gray matter [**GM**] and white matter [**WM**] tissue compositions at each voxel). The PSF derives from use of a finite number of k-space samples when acquiring spectroscopic data, and from smoothing the data with a Hamming window prior to spatial reconstruction. We therefore estimated the PSF by simulating[34] within k-space the acquisition of MRS data on a 24x24 grid and then using a Hamming window to spatially filter the simulated data. The full width at half maximum (**FWHM**) of the PSF equaled 30 mm. We interpolated the 24x24 complex array to 256x256 so as to match the spatial resolution of the T1w MR images. We estimated the proportions of GM and WM at each MRS voxel by coregistering to the MRS data the corresponding high-resolution T1w image, along with its map of GM and WM definitions (see above), and then convolving the coregistered tissue definitions with the PSF to calculate the proportions of GM and WM within each MRS voxel. We used a linear regression model[35] to estimate at a voxel *i* the concentration of each metabolite *j* within gray matter ($M_{ij}^{G}$) and white matter ($M_{ij}^{W}$):

$$S_{ij}=|c_{i}^{G}*M_{ij}^{G}+c_{i}^{W}*M_{ij}^{W}|+n_{i}$$

where $c_{i}^{G}$ and $c_{i}^{W}$ are the proportions of GM and WM, respectively, at voxel *i* of the MRS dataset, *n*_*i*_ is noise, and *S*_*ij*_ is the concentration of the metabolite in voxel *i* and its neighboring voxels. We then tri-linearly resampled the metabolite concentrations $M_{ij}^{G}$ and $M_{ij}^{W}$ from the low resolution MRS data to the high resolution anatomical data during spatial normalization across study participants.

#### Spatial normalization of MPCSI data

We coregistered the MPCSI data for each participant into the coordinate space of a single T1w template brain. We selected the template brain using a two-step procedure described elsewhere.[36] Briefly, we selected as a preliminary template the brain of a person who was demographically representative of all study participants, and then we nonlinearly warped the T1w image of each participant brain to that initial template. Next, from the participant brains warped to the initial template, we selected as the final template the brain that was morphologically closest in the least squares sense to the average brain across all participants. We then repeated coregistration and nonlinear warping of all brains to the final template brain.

We next coregistered each metabolite image into the coordinate space of the template brain, as follows. First, we coregistered each participant localizer image to its corresponding high-resolution T1w image using a similarity transform (3 translations, 3 rotations) that maximized mutual information[37] across the localizer and its corresponding T1w image. Next, we spatially transformed the coregistered localizer using the similarity transform that coregistered the participant T1w image into the coordinate space of the template. Finally, we warped the coregistered localizer by applying the same high-dimensional, nonlinear deformation that had warped the participant’s T1w image to the T1w template. We applied these transformations in the same order to the participant’s metabolite maps for normalizing them into the template space.

#### Normalizing low-resolution MRS data into high-resolution anatomical MRI space

The spatial normalization faithfully preserved the MRS data into the high-resolution anatomical MRI space, as our previously published study demonstrated that the region-of-interest based findings matched the voxelwise findings.[38] We normalized the low-resolution MRS data into the high-resolution anatomical space for the following two reasons. (1) We wanted to generate within-individual metabolites maps that were adjusted for the partial volume of various brain tissues as an MRS voxel at a 1000 mm^3^ resolution contains varying amounts of gray matter (GM), white matter (WM), and cerebrospinal fluid (CSF). Metabolite concentrations differ by tissue type[39–41] and because MRS signal is a weighted sum of signals from various tissue types, using the average metabolite signal from an MRS voxel will reduce sensitivity for detecting abnormalities and changes in metabolite concentrations. We corrected for the effects of partial volumes of different tissue types in each MRS voxel by first segmenting brain tissue in high-resolution anatomical MRI and then using the segmented tissue maps for computing the amount of each tissue in every MRS voxel. The MRS data and tissue maps in neighboring MRS voxels were used subsequently for estimating the MRS signal from each tissue type. We then assigned the estimated MRS signal for GM (or WM) to all GM (or WM) voxels within that MRS voxel at the high-resolution anatomical MRI space. Partial-volume correction and assignment of the tissue-specific MRS signal, therefore, generated maps of brain metabolites that were anatomically accurate, adjusted for partial volumes of tissue types, and contiguously distributed across the entire brain. (2) We wanted to quantify metabolite concentrations within homologous brain regions across individuals. Homologous brain regions cannot be precisely delineated in the low-resolution MRS space because of the differing head positions across participants. Although we followed a detailed protocol that carefully placed the 6 MRS slabs at predefined anatomical locations through the brain, across participant variability in head position and head size inevitably leads to variability in the placement of those slabs. An MRS slab, and an MRS voxel with that slab, therefore may contain signal from different brain regions across individuals. Furthermore, variability in brain morphology across participants leads to differing amounts of GM, WM, and CSF even in MRS voxels in roughly the same brain region. That is, across individuals, an MRS voxel will span different brain regions and will comprise different amounts of brain tissues. Normalization of MRS data into the high-resolution anatomical space permitted us to compare and analyze MRS data from homologous regions across individuals.

### Outcomes

Primary outcome measures were concentrations of NAA. Secondary outcome measures were concentration of other important brain metabolites, including glutamate+glutamine (Glx), creatine (Cr), and choline (Ch).

### Statistical analyses

We conducted primary analyses using baseline and longitudinal data to test our *a priori* hypothesis of treatment effects on NAA concentrations across voxels that had valid MRS data in at least half the patients and half the healthy controls (**S2 Fig**). Subsequently, we conducted secondary analyses assessing whether NAA mediated medication effects on symptom change. Finally, to understand better the cellular and physiological determinants for the changes in NAA concentrations, we conducted tertiary analysis of Ch, Cr, and GLX concentrations (**S1 File**). Our primary hypothesis was that NAA levels would differ significantly in patients relative to controls at baseline, and that NAA concentrations would normalize following treatment with duloxetine over the 10-week trial. Repeated measures in two groups each with 20 participants provides 80% statistical power at two-sided significance level of 0.05 to detect a medium to large change in the difference of group means.[42] Our dependent variable was NAA concentrations normalized by background noise. We normalized by background noise for two reasons: (1) it controlled for scanner drift, given that variation in transmitter and receiver gain affects metabolite concentrations and background noise equally; (2) use of NAA concentration relative to Cr concentrations in longitudinal analyses can lead to findings that are inscrutable, because both numerator and denominator values change over time, and those changes differ across treatment arms. We covaried for age and sex, and applied a procedure[43] for False Discovery Rate (**FDR**) to control for false positive (i.e., Type 1 error) when conducting multiple hypotheses testing across all voxels in the brain (Statistical Analyses, **S1 File**). We conducted baseline, repeated measures, and longitudinal mediation analyses to understand whether patients relative to healthy controls had abnormal NAA concentration at baseline, whether those levels normalized in patients treated with antidepressant medications, and whether normalization of NAA concentrations mediated the treatment effects on symptom severity.

#### Baseline analyses

We assessed whether NAA concentrations in patients differed from those in controls by applying multiple linear regression: *y*_*i*_ = *β*_0_+*β*_1_∙Age_*i*_+*β*_2_∙Sex_*i*_+*β*_3_∙Dx_*i*_+*ϵ*_*i*_, where diagnosis Dx_*i*_ = 0 for healthy participants, Dx_*i*_ = 1 for patients, and *y*_*i*_ is the NAA concentration in the *i*^th^ patient. We also assessed whether differences in NAA concentrations were proportionally greater in patients with more severe symptoms (Sx): Sx_*i*_: *y*_*i*_ = *β*_0_+*β*_1_∙Age_*i*_+*β*_2_∙Sex_*i*_+*β*_3_∙Sx_*i*_+*ε*_*i*_.

#### Longitudinal data analyses

We used a linear mixed model[44] to assess the effects of the treatment-by-time interaction on NAA concentrations–i.e., whether concentrations changed differentially across the two treatment arms: *y*_*ij*_ = *β*_0_+*β*_0*i*_+*β*_1_∙Age_*i*_+*β*_2_∙Sex_*i*_+*β*_3_∙Time_*ij*_+*β*_4_∙Tx_*i*_+*β*_**5**_∙Time_*ij*_∙Tx_*i*_+*ε*_*ij*_, where *y*_*ij*_ is metabolite concentration in the *i*^th^ patient at time *j* (*j* = 0 at baseline, 1 at week 10), Tx_*i*_ is treatment assignment in the *i*^th^ patient (Tx_*i*_ = 0 if treated with placebo, 1 with medication), Age_*i*_ is age at baseline, and *ε*_*ij*_ is within-individual measurement error, assuming a compound symmetry covariance matrix. We expected metabolite levels to change differently between the 2 treatment arms, and therefore, we tested this a priori hypothesis using the *β*_**5**_∙Time_*ij*_∙Tx_*i*_ interaction. In a post hoc analysis to understand this interaction better, we assessed change in metabolite levels separately in each treatment arm using the linear mixed model *y*_*ij*_ = *β*_0_+*β*_0*i*_+*β*_1_∙Age_*i*_+*β*_2_∙Sex_*i*_+*β*_3_∙Time_*ij*_+*ε*_*ij*_.

#### Repeated measures analyses with symptom severity

We assessed how the change in NAA concentrations correlated with change in symptom severity in the duloxetine-treated patients. Our previous studies[45, 46] showed that symptom severity decreased significantly in duloxetine-treated patients. Therefore, in an additional *post-hoc* analysis, we used a repeated measures analysis[47] to assess how within-individual change in symptom severity associated with changes in NAA levels: $y_{ij}=\beta_{0}+\beta_{0i}+\beta_{1}∙{Age}_{i}+\beta_{2}∙{Sex}_{i}+\beta_{3}∙{Time}_{ij}+\beta_{4}∙{\bar{Sx}}_{i}+\beta_{5}∙({Sx}_{ij}-{\bar{Sx}}_{i})+\varepsilon_{ij}$, where ${\bar{Sx}}_{i}=\frac{1}{2}({Sx}_{i1}+{Sx}_{i2})$ is the average of symptom severity at baseline (Sx_*i*1_) and end point (Sx_*i*2_) for the *i*^th^ patient, *β*_4_ is the between-individual correlation of NAA levels with severity, and *β*_5_ is the longitudinal, within-individual change in NAA levels accompanying the change in severity.

#### Longitudinal mediation analysis

We conducted *post hoc* mediation analyses to evaluate the causal relation between treatment and changes in NAA concentrations and symptom severity. If group differences in concentrations are pathogenic, then normalization of concentrations should mediate the treatment effects on symptom severity. However, if differing concentrations are instead a response to the presence of illness, then changes in symptom severity should mediate the treatment effects on NAA concentrations. We therefore tested each of these competing hypotheses. We show here the models for a two-wave mediation analysis[48–50] that tested the second of these hypotheses.

$$y_{i,2}=\beta_{0,1}+\beta_{1,1}∙{Age}_{i}+\beta_{2,1}∙{Sex}_{i}+c_{1}∙{Tx}_{i}+b_{1}∙{Sx}_{i,1}+b_{2}∙{Sx}_{i,2}+g_{1}∙y_{i,1}+\varepsilon_{i,1}$$

$${Sx}_{i,2}=\beta_{0,2}+\beta_{1,2}∙{Age}_{i}+\beta_{2,2}∙{Sex}_{i}+a_{1}∙{Tx}_{i}+g_{2}∙{Sx}_{i,1}+\varepsilon_{i,2}$$

where Sx_*i*,1_ and Sx_*i*,2_ are symptom severity and *y*_*i*,1_ and *y*_*i*,2_ are metabolite levels at baseline and week 10, respectively, for the *i*^th^ patient. We used the Sobel test[51] to assess the cross-time mediated effects of symptom severity, testing the statistical significance of the product *a*_1_*b*_1_, with its standard error (**se**) computed as ${se}_{ab}=\sqrt{\left(a_{1}^{2}∙{se}_{b}^{2})+(b_{1}^{2}∙{se}_{a}^{2}\right)}$, where *se*_*a*_, *se*_*b*_ are the standard errors of the coefficients *a*_1_ and *b*_1_, respectively. An analogous model tested the alternative hypothesis that change in metabolite levels mediated the effects of treatment on change in symptom severity.

### Role of the funding source

The funder of the study had no role in the study design, data collection, data analysis, data interpretation, or writing of the report. The corresponding author had full access to all the data in the study and had final responsibility for the decision to submit for publication.

## Results

Behavioral outcomes at baseline and at the end of the trial are detailed in our previously published clinical study.[45] Symptom severity decreased significantly during the trial in both arms (placebo probability value, p = 5.76*10^−3^; duloxetine p = 2.51*10^−15^); however, the decline in symptom severity was significantly greater (p = 8.54*10^−6^) in duloxetine-treated than in placebo-treated patients.[26]

### Baseline

**Fig 3:**
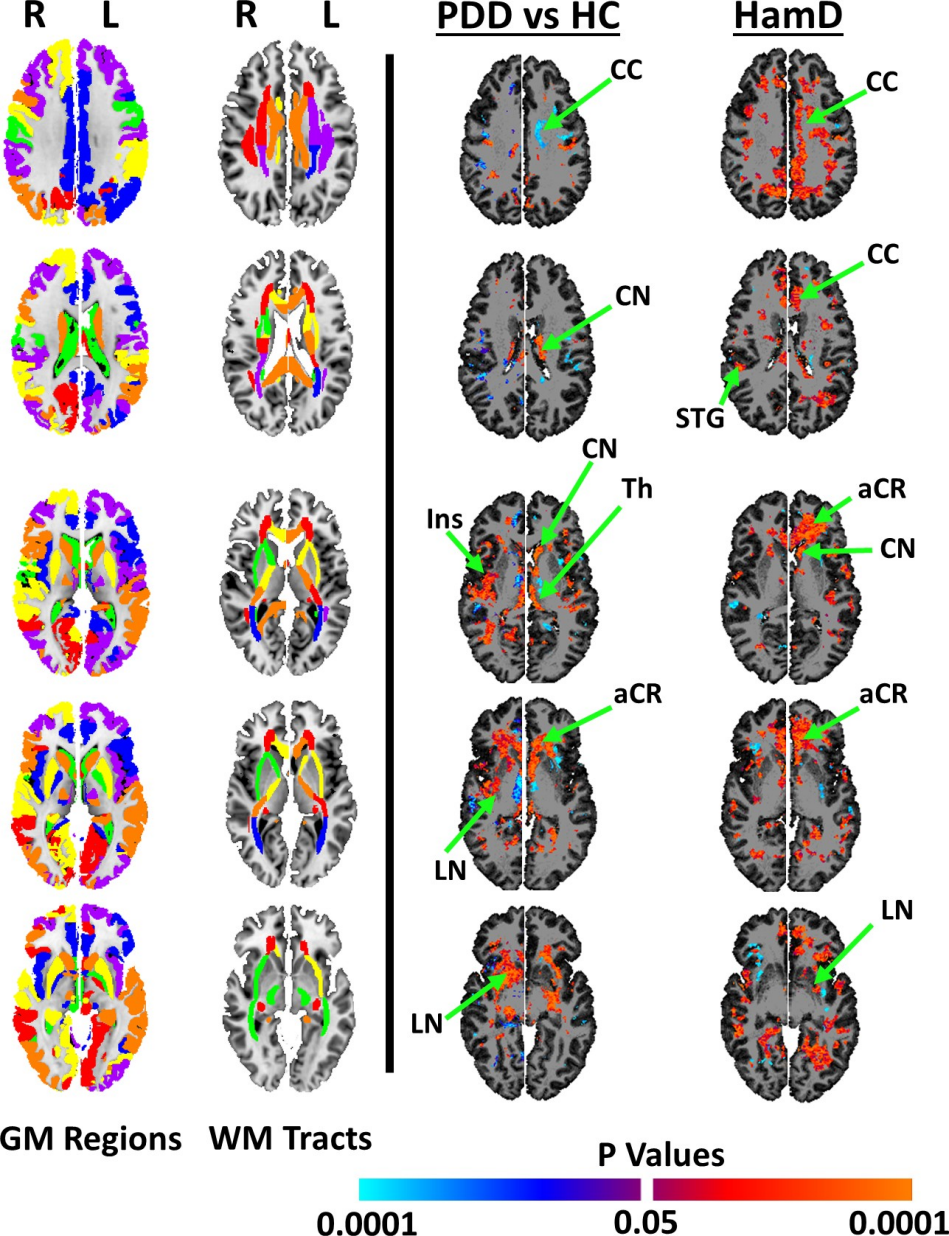
Baseline abnormalities in NAA concentrations and associations with symptom severity. At baseline, we compared NAA concentrations in 41 patients with dysthymic disorder (**PDD**) relative to 29 healthy controls (*Dx Effects*, *left column*) and correlated levels with symptom severity in the 41 patients (*HamD*, *right column*). The major white matter (**WM**) fiber pathways labeled in the Johns Hopkins atlas[65] (*WM Tracts*) were used to localize WM findings; the gray matter (**GM**) gyri labeled in the CCB atlas[66] (*GM Regions*) were used to localize GM findings. The WM atlas and the GM atlas were each normalized within the coordinate space of the template brain, thereby allowing us to localize precisely the findings to specific brain regions and WM fiber pathways. The findings are shown on 5 representative slices through the brain. **Dx Effects:** Patients, relative to controls, had higher metabolite concentrations across large portions of the brain, especially in the caudate nucleus (CN), anterior corona radiata (aCR), corpus callosum (CC), thalamus (Thal), right lenticular nucleus (putamen and globus pallidus), and posterior corona radiata (pCR). **HamD Correlations:** Symptom severity, measured using the Hamilton Depression Rating Scale (**HDRS**), correlated positively with NAA concentrations (i.e., patients with higher metabolite concentrations had more severe symptoms), especially in the sCR and CN. Transverse brain slices are shown in the radiological orientation. We controlled for false positives in all analyses using a false discovery rate (**FDR**) procedure and covaried for age and sex. We subsequently applied a cluster threshold that suppressed all findings of spatial extent smaller than 100 contiguous voxels. P-values are color coded, with positive associations displayed in warm colors (orange and red) and inverse associations in cool colors (cyan and blue). **Abbreviations: R**, Right Hemisphere; **L,** Left Hemisphere; **NAA**, N-acetyl Aspartate; **CC**, corpus callosum; **sCR**, superior corona radiata; **CN**, caudate nucleus; **aCR**, anterior corona radiata; **Ins**, insular; **Th**, thalamus; **LN**, lenticular nucleus (putamen and globus pallidus); **pCR**, posterior corona radiata.

NAA concentrations were higher in patients than controls in large portions of deep WM, especially in inferior frontal regions traversed by the anterior corona radiata (**aCR**). Confirmatory analyses at the resolution of MRS data acquisition showed that patients relative to healthy controls had higher NAA concentrations in the inferior frontal regions. Concentrations were also elevated in the caudate nucleus (**CN**), lenticular nucleus (**LN**), insula (**Ins**), and thalamus (**Th**). Concentrations were lower in WM of the corpus callosum (**CC**) **(Fig 3, left column)**.

NAA concentrations within patients correlated positively with symptom severity in regions where metabolite levels were higher in patients than in controls (**Fig 3, right column),** indicating that patients with higher NAA concentrations had more severe symptoms.

### Changes during the RCT

**Fig 4:**
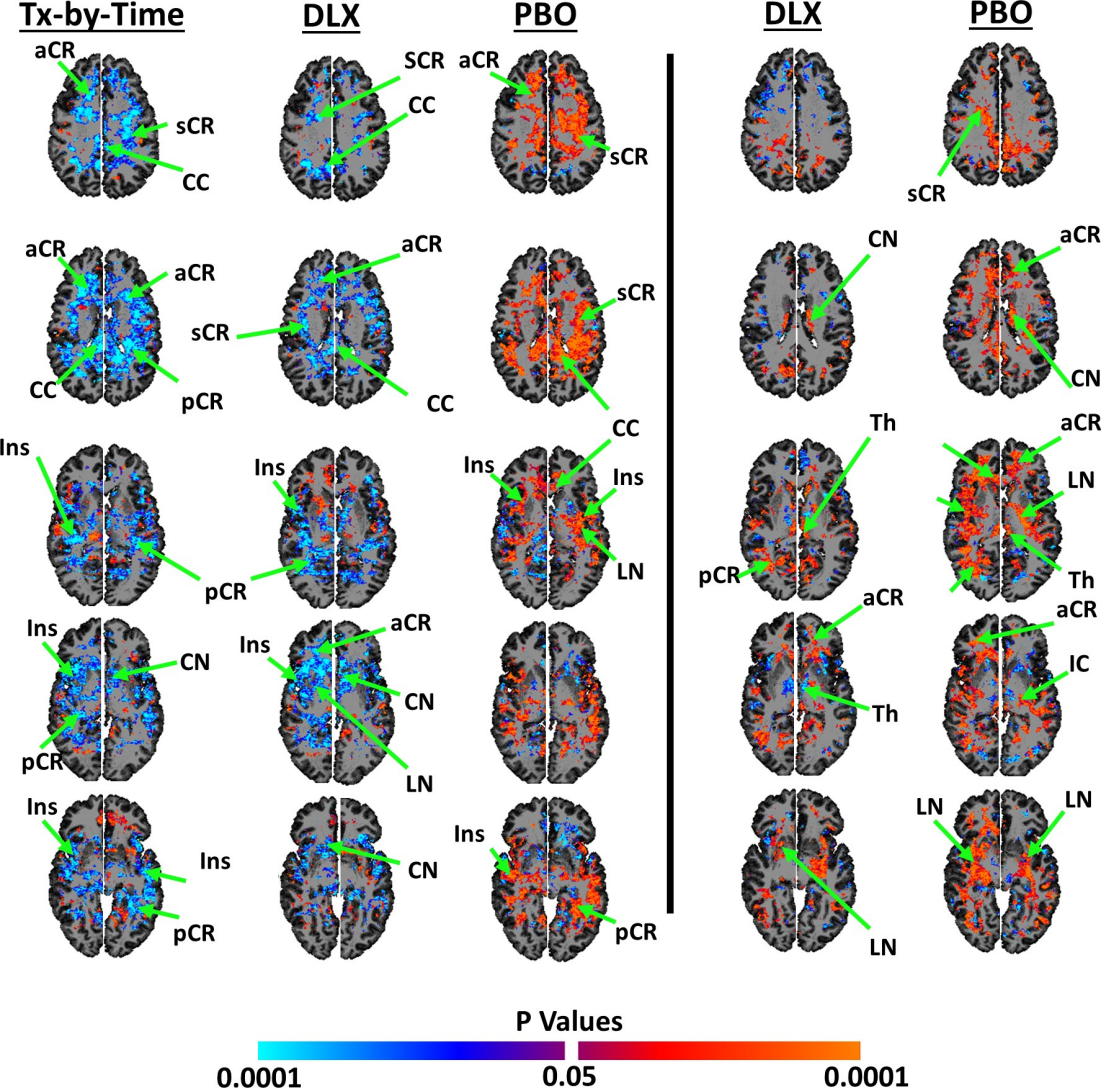
Changes in NAA concentrations over the 10-Week trial. *Left Panel*: We applied repeated measures analyses to assess whether NAA concentrations in duloxetine-treated patients changed differently than those in placebo-treated patients. The analyses showed significant treatment-by-time effects on metabolite levels: that is, metabolite levels in duloxetine-treated patients changed differently than those in placebo-treated patients across large portions of the brain. We therefore assessed separately in duloxetine-treated (*DLX*) and in placebo-treated (*PBO*) patients how NAA concentrations changed during the clinical trial. These analyses showed that NAA concentrations in duloxetine-treated patients declined significantly across large portions of the brain, whereas in placebo-treated patients concentrations increased across large portions of the brain. *Right Panel*: At the end of the trial, we assessed separately how NAA concentrations in duloxetine-treated or in placebo-treated patients differed from those in healthy controls. Concentration levels were normalized across large portions of the brain in duloxetine-treated (*DLX*) but increased and deviated further away from healthy values on placebo-treated patients (*PBO*). P-values in all analyses that survived the FDR procedure for multiple comparisons and a cluster-level threshold of 100 contiguous voxels were color encoded using warm colors (orange and red) for increased and cool colors (cyan and blue) for decreases in NAA concentrations. **Abbreviations: R**, Right Hemisphere; **L,** Left Hemisphere; **NAA**, N-acetyl Aspartate; **CC**, corpus callosum; **sCR**, superior corona radiata; **CN**, caudate nucleus; **aCR**, anterior corona radiata; **Ins**, insular, **CC**, corpus callosum, **Th**, thalamus; **LN**, lenticular nucleus (putamen and globus pallidus); **pCR**, posterior corona radiata; **IFG**, inferior frontal gyrus.

NAA concentrations changed differentially across the two groups during the trial (**Fig 4, left panel**). *Post hoc* analyses showed that duloxetine treatment significantly normalized NAA concentrations toward control values, reducing concentrations in widely distributed WM regions (the Superior Corona Radiata, **sCR**; Posterior Corona Radiata, **pCR**; aCR; **CC**). Duloxetine treatment also reduced concentrations in GM (CN, LN, Th, Ins) (**Fig 4, left panel**). In contrast, NAA concentrations in placebo-treated patients increased significantly during the trial, even further from control values, across widely distributed WM (**Fig 4, right panel**) and GM regions. The treatment effects did not alter when we covaried for line width of fitting or education level of patients.

**Fig 5:**
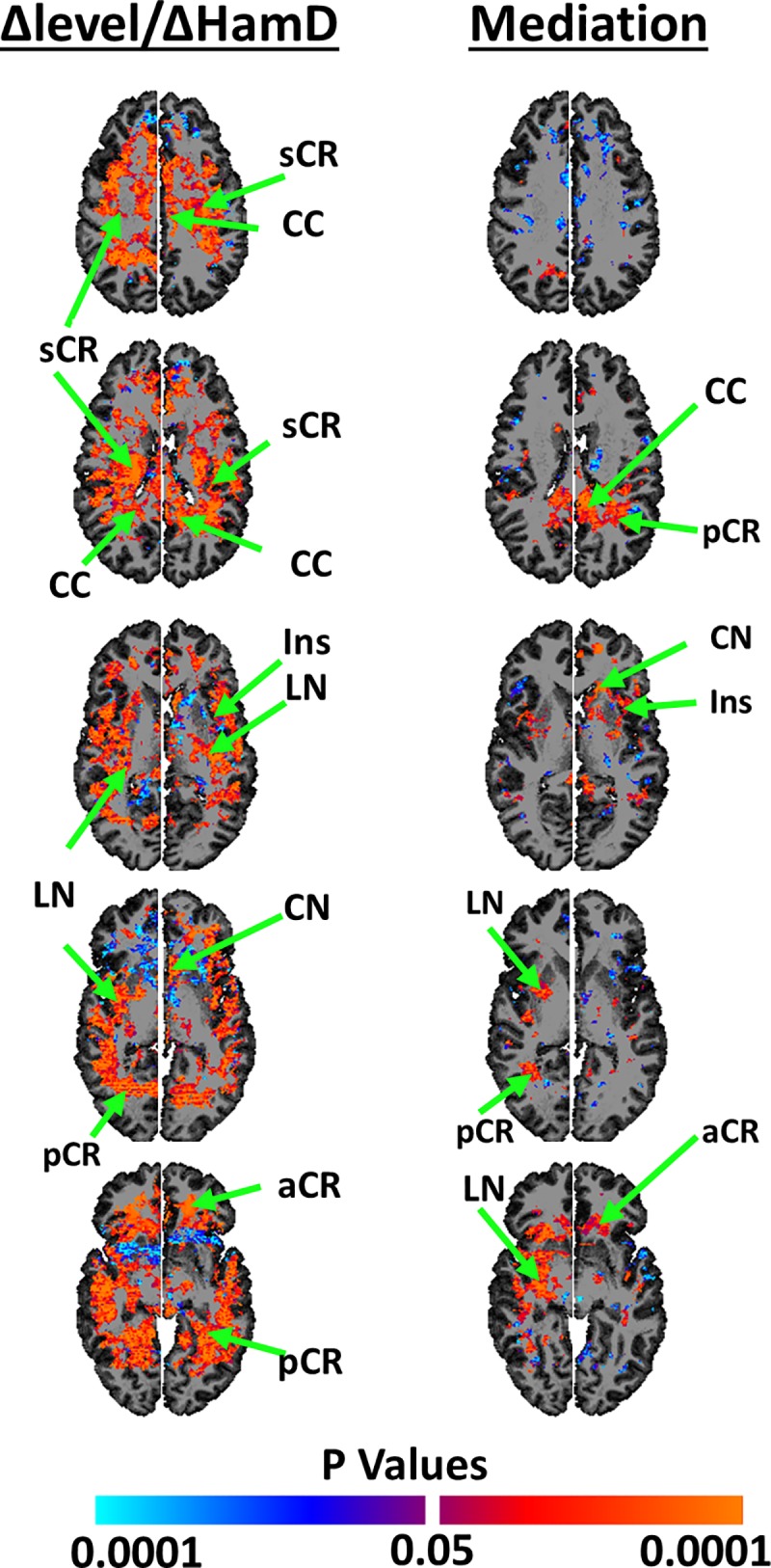
Changes in NAA concentrations and symptom severity. *Left Column*: Using repeated measures analyses, we assessed within duloxetine-treated patients how NAA concentrations changed with changes in symptom severity. These analyses showed that in general the change in NAA concentrations were positively associated with the change in symptom severity: i.e., NAA concentrations decreased towards healthy values as symptom severity decreased in duloxetine-treated patients. *Right Column*: We subsequently applied longitudinal mediation analyses to assess whether changes in symptom severity mediated the treatment effects on changes in NAA concentration. These analyses showed that symptom severity significantly mediated the change in NAA concentrations as a consequence of treatment in the caudate nucleus (**CN**) and putamen (**PUT**). Mediation analyses provided no statistical evidence for the alternate hypothesis, that the change in NAA concentrations mediated the treatment effects on change in symptom severity. All maps are FDR-corrected at p<0.05, and analyses included age and sex as covariates. We subsequently applied a cluster threshold that suppressed all findings of spatial extent smaller than 100 contiguous voxels. Positive associations were coded in warm colors (orange and red); inverse associations were coded in cool colors (cyan and blue). **Abbreviations: R**, Right Hemisphere; **L,** Left Hemisphere; **NAA**, N-acetyl Aspartate; **CC**, corpus callosum; **sCR**, superior corona radiata; **CN**, caudate nucleus; **aCR**, anterior corona radiata; **Ins**, insular, **Th**, thalamus; **LN**, lenticular nucleus (putamen and globus pallidus); **pCR**, posterior corona radiata.

Repeated-measures analyses within duloxetine-treated patients alone showed that the change in NAA concentrations correlated positively with the change in symptom severity across most of the brain, including in WM regions of the aCR, sCR, pCR, CC, and in GM regions of CN, LN, Th, Ins. In other words, NAA concentrations declined in these regions as symptom severity declined over the 10-week trial (**Fig 5, left column**).

Mediation analyses showed that the change in symptom severity significantly mediated treatment effects on change in NAA concentration in WM regions of the CC, sCR, pCR, and in GM regions of CN, right LN, Th (**Fig 5, right column)**. The opposing hypothesis, that NAA concentrations mediated the effects of treatment on symptom severity, was not statistically significant in any brain region.

## Discussion

As we hypothesized, baseline NAA concentrations differed significantly at baseline in patients from healthy controls, with significant elevations present in both WM and subcortical GM. Concentrations at baseline correlated positively with symptom severity, indicating that greater NAA concentrations accompanied more severe symptoms. Concentrations also correlated inversely with symptoms in several brain regions, suggesting that in those regions altered levels may have been compensatory. At the end of the trial, concentrations in many regions had normalized in duloxetine-treated patients, whereas they either remained elevated or increased even further from healthy values in placebo-treated patients. Finally, mediation analyses showed that the reduction in symptom severity mediated the effects of treatment on the change in NAA concentrations, especially in WM of the frontal lobe and corpus callosum, and in the caudate, putamen, and insula. We did not detect evidence that changes in NAA concentrations mediated treatment effects on the improvement of depressive symptoms. These findings taken together suggest that, as hypothesized, duloxetine administration normalized NAA concentrations in patients, but that it did so by modulating the severity of depressive symptoms. Medication administration presumably reduced depressive symptoms through other, as yet unidentified, brain processes.

### Baseline findings

At baseline, NAA concentrations in patients relative to healthy controls were (a) higher in WM pathways of the anterior corona radiata (aCR), superior corona radiata (sCR), posterior corona radiata (pCR), and corpus callosum (CC); (b) lower in WM pathways of the corpus callosum (CC); (c) higher in GM of the caudate nucleus (CN), right lenticular nucleus (LN), thalamus (Th) and insula (Ins). Thus, NAA concentrations were altered in major WM pathways, including cortical-subcortical fibers projecting through the anterior and posterior corona radiata and the corpus callosum supporting interhemispheric communication. The aCR, on the other hand, interconnects cortical regions with the subcortical gray matter nuclei of the CN, LN, and Th to form cortico-striato-thalamo-cortico (CSTC) circuits that support the regulation of cognitive, emotional, and motor programs.[52, 53] As NAA is thought to be a marker for the density of healthy neurons,[4] the higher NAA concentrations in CSTC circuits in PDD patients compared with controls at baseline, and in direct proportion to the severity of symptoms, suggest an attempt to compensate for the presence of depressive symptoms, presumably through engagement of regulatory systems to control or contain symptoms, perhaps even preventing manifestation of a full-blown major depressive disorder. The reduced NAA concentrations in the CC suggest a reduced density of healthy neurons in the CC and perhaps impaired communication among homologous regions of the two hemispheres in this chronic illness. These abnormal NAA concentrations in direct proportion to the symptom severity suggest that WM abnormalities may contribute to the genesis of symptoms in PDD patients.

NAA concentrations also were abnormal in several GM regions that support emotional and cognitive processes, including (**a**) the caudate, LN, and thalamus which, in addition to regulating the planning and sequencing of motor tasks,_ regulate working memory,[54] motivation, and reward perception;[55] and (**b**) the insula, which supports subjective emotional experience, emotional processing,[56] empathy, and social decision-making.[57] Thus, our findings may point to a neurophysiological substrate for disrupted cognitive and emotional processing in PDD.

The widely distributed findings at baseline suggest that the circuit-based substrates of PDD are equally diverse and widespread. Nevertheless, the regions with the most prominent elevations in NAA concentrations at baseline were in frontal WM, especially inferior regions, and the basal ganglia and thalamus. These regions are widely known to support, among other things, emotional processing, hedonic reward, cognitive flexibility, and the regulation of impulses and emotions.

### Treatment-specific changes

Over the 10-week RCT, NAA concentrations normalized in most regions in duloxetine-treated patients, whereas they either remained unchanged or deviated further from control values in placebo-treated patients. Mediation analyses provided strong evidence that medication-induced reductions in symptom severity mediated the medication-induced reductions in NAA concentrations, especially in frontal WM, caudate, putamen, and insula. In other words, duloxetine attenuated the severity of symptoms, which in turn normalized NAA concentrations likely because of the reduced need for compensatory control; in contrast, medication-induced changes in NAA concentrations did not significantly mediate medication-induced changes in symptom severity. We likely detected mediation effects in frontal WM, caudate, and putamen because these brain regions compose a critically important set of neural circuits that project dense glutamatergic transmission to and from the cortex, basal ganglia, and thalamus,[58] thereby conferring sufficient treatment-induced changes in MRS signal and adequate statistical power to detect mediation effects.

Even though the RCT demonstrated conclusively that duloxetine modulated NAA concentrations and even normalized them relative to control values, mediation analyses suggested that depression is likely not caused by an imbalance of these metabolites in the brain; rather, these metabolite imbalances are likely a consequence of having depression, and their concentrations deviate from control values in direct proportion to the severity of depressive symptoms. Duloxetine likely is changing depressive symptoms through some other, as yet unidentified, brain mechanism, not through the normalizing of metabolite concentrations.

### Relation to prior imaging studies of depressive illness

Our findings of higher NAA, Cr, and Glx concentrations in PDD patients differ from the lower concentrations of these metabolites that have generally been reported previously in patients with MDD.[15] They also disagree with findings of a case-control, 12-week clinical trial of duloxetine in treatment-naïve adults with first-episode MDD, which reported that NAA/Cr and Ch/Cr ratios in patients at baseline did not differ from those in healthy controls, suggesting that metabolite levels are unaltered in the early stages of MDD.[59] The use of ratios to Cr may well be responsible for the non-findings in that study, as Cr, NAA, and Ch concentrations are intercorrelated. In addition, studies of blood perfusion in the brain, an index for cellular metabolism, using MRI-based arterial spin labeling (ASL) techniques have largely reported hypoperfusion in the default mode network and limbic cortices in patients with MDD relative to healthy controls.[60] Hyperperfusion, however, has also been reported in the subcallosal cingulate, putamen, and fusiform gyrus.[61] Hyperperfusion also has been reported in patients with chronic, treatment-resistant depression in the subgenual anterior cingulate cortex, ACC, and left subcortical regions.[62] Studies using single photon emission computed tomography have also reported hyperperfusion in depressed patients relative to patients with cognitive disorder,[63] and in the cerebellum of PDD patients relative to healthy controls.[64]

Of course, one possible explanation for the discrepant findings between PDD and MDD, despite their common clinical phenotype, is that the two conditions may differ in their pathogenesis and biological signatures, especially as they have differing chronicities. MDD is episodic, and the lower metabolite concentrations and hypoperfusion reported in MDD may be a state marker for acute illness. PDD, however, is a chronic illness of at least 2 years duration, which could in some way create a hypermetabolic state in proportion to the severity of depressive symptoms. Speaking against this potential explanation, however, is the relatively rapid change in NAA concentrations observed over the course of this RCT, which shows that clinical improvement or remission of less than 10 weeks duration is sufficient to alter metabolite concentrations. Alternatively, the presence of neuroplastic compensation in persons with PDD may help to keep depressive symptoms from increasing to full-blown MDD,[26] and if neural plasticity is driving the increase in metabolite concentrations in PDD, it could explain the discrepant NAA findings in PDD and MDD, especially if neuroplastic compensation for some reason is absent in MDD. Finally, the discrepant findings could derive from the limitations of prior MRS studies of MDD, which include: the limited regional sampling that single voxel MRS affords; the often greater partial volume effects that accompany use of a larger voxel (typically >8 cm^3^) in single voxel MRS; and the use of metabolite ratios, which could be destabilized if the metabolite concentrations are highly intercorrelated, as data from the present study suggests is the case.

### Limitations

Our findings should be interpreted in the light of the following limitations. First, we acquired MRS data using a long echo time (TE = 144 ms), which limited measurement to the more abundant brain metabolites—NAA, Cr, Cho, and Glx. However, the use of long TE also had the advantage of flattening the spectral baseline, thereby minimizing contamination of metabolite signals from macromolecules and improving the accuracy of spectral fitting for metabolite quantification. Second, saturation bands to suppress lipid signal from the scalp were not as precisely shaped as the scalp. These inaccuracies inevitably suppressed metabolite signals from several portions of cortical gray matter, and consequently MRS data for most participants were available primarily within WM and deep gray matter nuclei (**S2 Fig**). Third, 4 patients had prior exposure to psychotropic medications, which could conceivably have influenced our findings, though this seems unlikely, as those patients underwent a 4-week washout before entering the trial, and excluding them from analyses did not change our findings. Fourth, the sex composition of patients differed across the 2 treatment arms, with significantly more men in the placebo than duloxetine arm of the study. However, differing sex composition was unlikely to have confounded our findings because symptom severity did not differ between men and women at either baseline or end of the trial. Moreover, men had lower metabolite concentrations than women (**S7 Fig**), and therefore higher metabolite concentrations in the placebo arm at the end of the trial likely did not derive from a higher proportion of men in the placebo arm. Fifth, acquiring MRS data only once in healthy controls may have limited our interpretation of changes in NAA concentrations in placebo-treated patients. The second time point, for example, would have allowed us to determine whether the significant increase in NAA concentrations that we detected in the placebo-treated patients differed significantly from the change in NAA concentrations over time in heathy adults. That would have been useful, but it is not needed to know that NAA concentrations declined over time in response to medication.

## Conclusions

Our *a priori* and *post hoc* analyses showed that NAA concentrations were significantly elevated across numerous brain regions in direct proportion to symptom severity at baseline, and then normalized during treatment with medication. Mediation analyses indicated that treatment reduced symptom severity not by normalizing metabolite concentrations, but through other, as yet unknown, brain mechanisms. We therefore expect that other classes of anti-depressant drugs and possibly psychological and behavioral therapies that reduce symptom severity will also likely normalize NAA in the brain. Thus, abnormal NAA concentrations are likely secondary to a more fundamental pathophysiological process, such as altered bioenergetics, mitochondrial dysfunction, glial cell abnormalities, neuroinflammation, or some combination of them, given that they are highly interdependent, non-mutually exclusive processes.

## Supporting information

S1 Fig Example MRS spectrum and spectral fitting for quantifying metabolite concentrations. We show two example MRS spectrums (dark blue), spectral fitting for NAA (violet), Ch (dark red), Cr(green), Glx (light blue), and residual error (orange). These plots show that the acquired MRS data were of excellent quality, leading to accurate fitting of the various spectral peaks.
(TIF)

S2 Fig Map for the number of participants at each voxel with MRS data. We acquired MRS data by applying eight saturation bands to suppress lipid signal from the scalp, thereby preventing contamination of the metabolite signal in the brain. However, because the saturation bands cannot be placed precisely to the scalp, metabolite signal especially in the cortical mantel is either suppressed by the saturation bands or gets contaminated from the lipid signal. The MRS voxels with contamination are discarded and not included in statistical analyses. Furthermore, to ensure that data are available from a sufficient number of patients and healthy controls, we performed statistical analyses only on those voxels where MRS data were available for at least 20 patients and 15 healthy controls. *Top Row*: grayscale map of the number of participants at voxels with MRS data for at least 20 patients and 15 healthy controls. Nonbrain voxels or voxels with MRS data from fewer patients or healthy controls are shown in black. *Bottom Row*: the cyan contour on the template brain is the boundary of this region for visualizing brain regions where MRS data is available for at least 20 patients and 15 healthy controls.
(TIF)

S3 Fig Voxelwise maps of average metabolite concentrations. Using baseline MRS data normalized into the coordinate space of a template brain, we generated voxelwise map for the average metabolite concentrations separately in patients (*Left Panel*) and in healthy controls (*Right Panel*). The metabolite concentrations were color coded and displayed only in brain regions where we had MRS data for at least half of patients and half of healthy controls. We color encoded concentration values with green denoting 0 and red denoting the maximum value for each metabolite: 400 for NAA, 225 for CH, 200 for CR, and 125 for GLX–i.e., the same color across metabolites maps different concentration value. We generated these color maps to compare visually the average metabolite concentrations in patients with those in healthy controls.
(TIF)

S4 Fig Baseline abnormalities in and associations of metabolites concentrations with symptom severity. At baseline, we separately compared Ch, Cr, and Glx concentrations in 41 patients with dysthymic disorder (**PDD**) relative to 29 healthy controls (*Dx Effects*). We also correlated metabolite levels with symptom severity in the 41 patients (*HamD Correlations*). **Dx Effects:** Patients, relative to controls, had higher metabolite concentrations across large portions of the brain, especially in the caudate nucleus (CN), anterior corona radiata (aCR), thalamus (Thal), right lenticular nucleus (putamen and globus pallidus), and posterior corona radiata (pCR); and had lower concentration lower in the corpus callosum (CC), superior corona radiata(sCR), and insula (Ins). **HamD Correlations:** Symptom severity, measured using the Hamilton Depression Rating Scale (**HDRS**), correlated positively with all metabolite levels (i.e., patients with higher metabolite concentrations had more severe symptoms), especially in the sCR, CN, and pCR. Therefore, patients relative to controls had higher metabolite concentrations, especially in the inferior portions of the brain, and those with higher levels had more severe symptoms. Transverse brain slices are shown in the radiological orientation. We controlled for false positives in all analyses using a false discovery rate (**FDR**) procedure and covaried for age and sex. We subsequently applied a cluster threshold that suppressed all findings of spatial extent smaller than 100 contiguous voxels. P-values are color coded, with positive associations displayed in warm colors (orange and red) and inverse associations in cool colors (cyan and blue).
(TIF)

S5 Fig Baseline association of metabolite concentration and symptom severity. We generated scatterplots for visually assessing how metabolite concentrations were associated with symptom severity at a select region in the brain that survived FDR correction for multiple comparisons. Metabolite concentrations along the Y-axis are in arbitrary internal units normalized by the amount of background noise. Furthermore, metabolite concentrations are adjusted for differing age and sex across participants and corrected for partial volume effects from differing tissue composition within each MRS voxel. These plots show that positive associations of increasing metabolite concentrations with increasing symptom severity are not a consequence of outlying values in the data.
(TIF)

S6 Fig Baseline associations of metabolite concentration with symptom severity assessed using CDRS. We assessed symptom severity in patients using both the 24-item Hamilton Depression Rating Scale (**HDRS**)[31] and the Cornell Dysthymia Rating scale (**CDRS**).[32] These maps show that metabolite concentrations were positively associated with symptom severity measured using CDRS across most regions of the brain. All maps were FDR-corrected at a false discovery rate of 0.05, covarying for age and sex. We subsequently applied a cluster threshold that suppressed all findings of spatial extent smaller than 100 contiguous voxels.
(TIF)

S7 Fig Sex Differences in metabolite concentrations. We assessed how NAA, Ch, Cr, and Glx concentrations in males differed from those in females at baseline using our entire cohort of 41 patients and 29 healthy controls while covarying for age and diagnosis. These analyses showed that males had significantly lower levels of metabolites across several regions of the brain. We controlled for false positives using a false discovery rate (**FDR**) procedure. We applied a cluster-level threshold of 100 voxel and color encoded P-values such that brain regions with higher metabolite concentrations in males are displayed in warm colors (orange and red) and those with lower concentrations in males are displayed in cool colors (cyan and blue).
(TIF)

S8 Fig Scatterplots for changes in metabolite concentrations. Over the 10-week period of the clinical trial, changes in metabolite concentrations in the duloxetine arm differed from those in the placebo arm of the trial (**Fig 2**). To understand better those changes, we generated scatterplots for changes metabolite concentrations at a brain region with significant effects of *treatment-by-time* interaction on metabolite concentrations. Metabolite concentrations along the Y-axis are in arbitrary internal units normalized by the amount of background noise. Furthermore, metabolite concentrations are adjusted for the age and sex of participants and are corrected for partial volume effects from differing tissue composition within each MRS voxel. These scatterplots show that at baseline (i.e., *Time 1*) concentrations in patients randomized to the duloxetine arm did not differ significantly from those in patients randomized to the placebo arm. However, concentrations increased in placebo-treated patients (*brown*) but declined in duloxetine-treated patients (*blue*) by the end of the trial (i.e., *Time 2*).
(TIF)

S9 Fig Changes in metabolite concentrations and symptom severity. *Left Panel*: Using repeated measures analyses, we assessed within duloxetine-treated patients how concentrations of Ch, Cr, and Glx changed separately with change in their symptom severity. These analyses showed that in general the change in metabolite levels were positively associated with change in symptom severity: i.e., metabolite levels decreased towards healthy values as symptom severity decreased in duloxetine-treated patients. *Right Panel*: We subsequently applied longitudinal mediation analyses to assess whether changes in symptom severity mediated the treatment effects on changes in metabolite concentration. These analyses showed that symptom severity significantly mediated the change in Glx concentrations as a consequence of treatment in the caudate nucleus (**CN**). Mediation analyses provided no statistical evidence for the alternate hypothesis, that the change in metabolite concentrations mediated the treatment effects on change in symptom severity. All maps are FDR-corrected at p<0.05, and analyses included age and sex as covariates. We subsequently applied a cluster threshold that suppressed all findings of spatial extent smaller than 100 contiguous voxels. Positive associations were coded in warm colors (orange and red); inverse associations were coded in cool colors (cyan and blue).
(TIF)

S10 Fig Symptom severity significantly mediated the associations of treatment with NAA concentration. The scatter plot shows the associations among treatment, change in symptom severity, and change in the levels of NAA in the caudate nucleus, where the mediating effects of symptom severity were statistically significant. Treatment significantly decreased symptom severity in duloxetine-treated patients (p = 2.4 x 10^−4^). The decline in symptom severity correlated significantly with the decline in NAA concentrations (p = 0.014), and treatment correlated significantly with the decline in Glx levels (p = 0.048).* = P-value<0.05; ** = P-value<0.01; *** = P-value<0.001.
(TIF)

S11 Fig Baseline abnormalities in metabolites levels while covarying for Cr. We assessed how baseline concentrations of NAA, Ch, and Glx in 41 patients differed from those in 29 healthy controls while covarying for age, sex, and Cr concentration. These analyses showed that metabolite concentrations in patients differed from those in healthy controls across the same brain regions and in the same direction as those in analyses without covarying for Cr (**Fig 1**, *left panel*). However, because Cr levels changed in the same direction as NAA and Glx levels and in the opposite direction as Ch levels, differences in NAA and Glx levels were attenuate whereas differences in Ch levels were accentuated. We controlled for false positives using a false discovery rate (**FDR**) procedure and covaried for age and sex. We subsequently applied a cluster threshold that suppressed all findings of spatial extent smaller than 100 contiguous voxels. P-values are color coded, with positive associations displayed in warm colors (orange and red) and inverse associations in cool colors (cyan and blue).
(TIF)

S1 File Supporting methods, results, and discussion. (DOCX)

S2 File CONSORT checklist. (DOCX)

S3 File Time 1 MRS Data for patients. (ZIP)

S4 File Time 2 MRS Data for patients. (ZIP)

S5 File MRS data for healthy controls. (ZIP)

S6 File Study protocol. (PDF)
