# Supplementary figures and images for: Effects of the antidepressant medication duloxetine on brain metabolites in persistent depressive disorder: A randomized, controlled trial
Source: PLoS One. 2019 Jul 19;14(7):e0219679. doi: 10.1371/journal.pone.0219679 (PMC6641507; doi:10.1371/journal.pone.0219679)

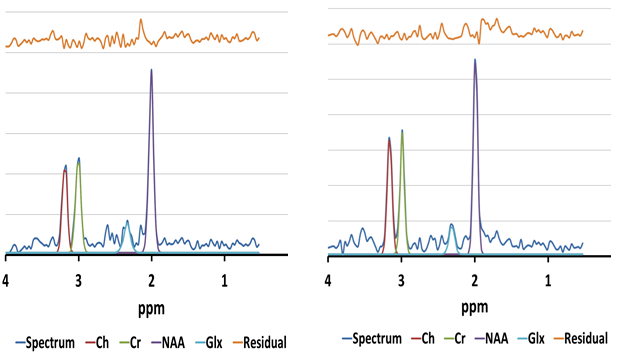

Supplement: S1 Fig — We show two example MRS spectrums (dark blue), spectral fitting for NAA (violet), Ch (dark red), Cr(green), Glx (light blue), and residual error (orange). These plots show that the acquired MRS data were of excellent quality, leading to accurate fitting of the various spectral peaks. (TIF) [file pone.0219679.s001.TIF]

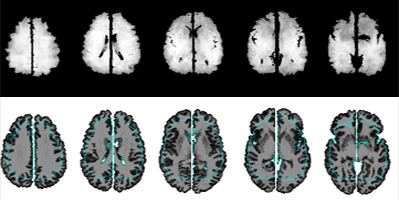

Supplement: S2 Fig — We acquired MRS data by applying eight saturation bands to suppress lipid signal from the scalp, thereby preventing contamination of the metabolite signal in the brain. However, because the saturation bands cannot be placed precisely to the scalp, metabolite signal especially in the cortical mantel is either suppressed by the saturation bands or gets contaminated from the lipid signal. The MRS voxels with contamination are discarded and not included in statistical analyses. Furthermore, to ensure that data are available from a sufficient number of patients and healthy controls, we performed statistical analyses only on those voxels where MRS data were available for at least 20 patients and 15 healthy controls. Top Row: grayscale map of the number of participants at voxels with MRS data for at least 20 patients and 15 healthy controls. Nonbrain voxels or voxels with MRS data from fewer patients or healthy controls are shown in black. Bottom Row: the cyan contour on the template brain is the boundary of this region for visualizing brain regions where MRS data is available for at least 20 patients and 15 healthy controls. (TIF) [file pone.0219679.s002.TIF]

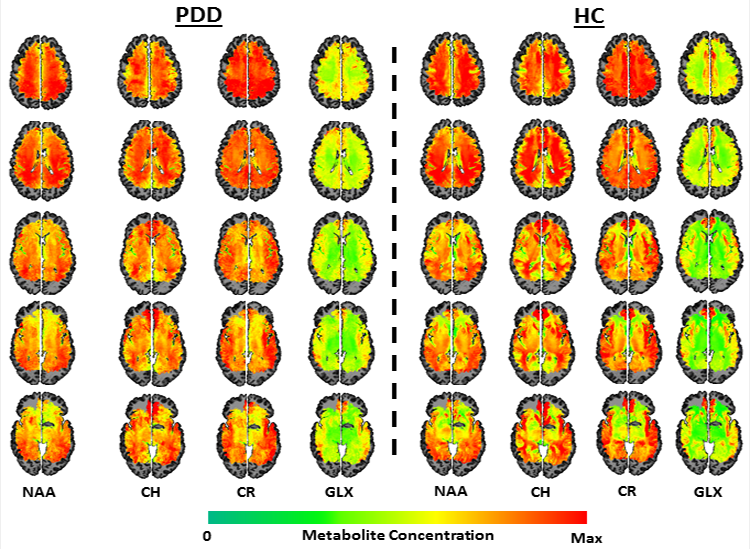

Supplement: S3 Fig — Using baseline MRS data normalized into the coordinate space of a template brain, we generated voxelwise map for the average metabolite concentrations separately in patients (Left Panel) and in healthy controls (Right Panel). The metabolite concentrations were color coded and displayed only in brain regions where we had MRS data for at least half of patients and half of healthy controls. We color encoded concentration values with green denoting 0 and red denoting the maximum value for each metabolite: 400 for NAA, 225 for CH, 200 for CR, and 125 for GLX–i.e., the same color across metabolites maps different concentration value. We generated these color maps to compare visually the average metabolite concentrations in patients with those in healthy controls. (TIF) [file pone.0219679.s003.TIF]

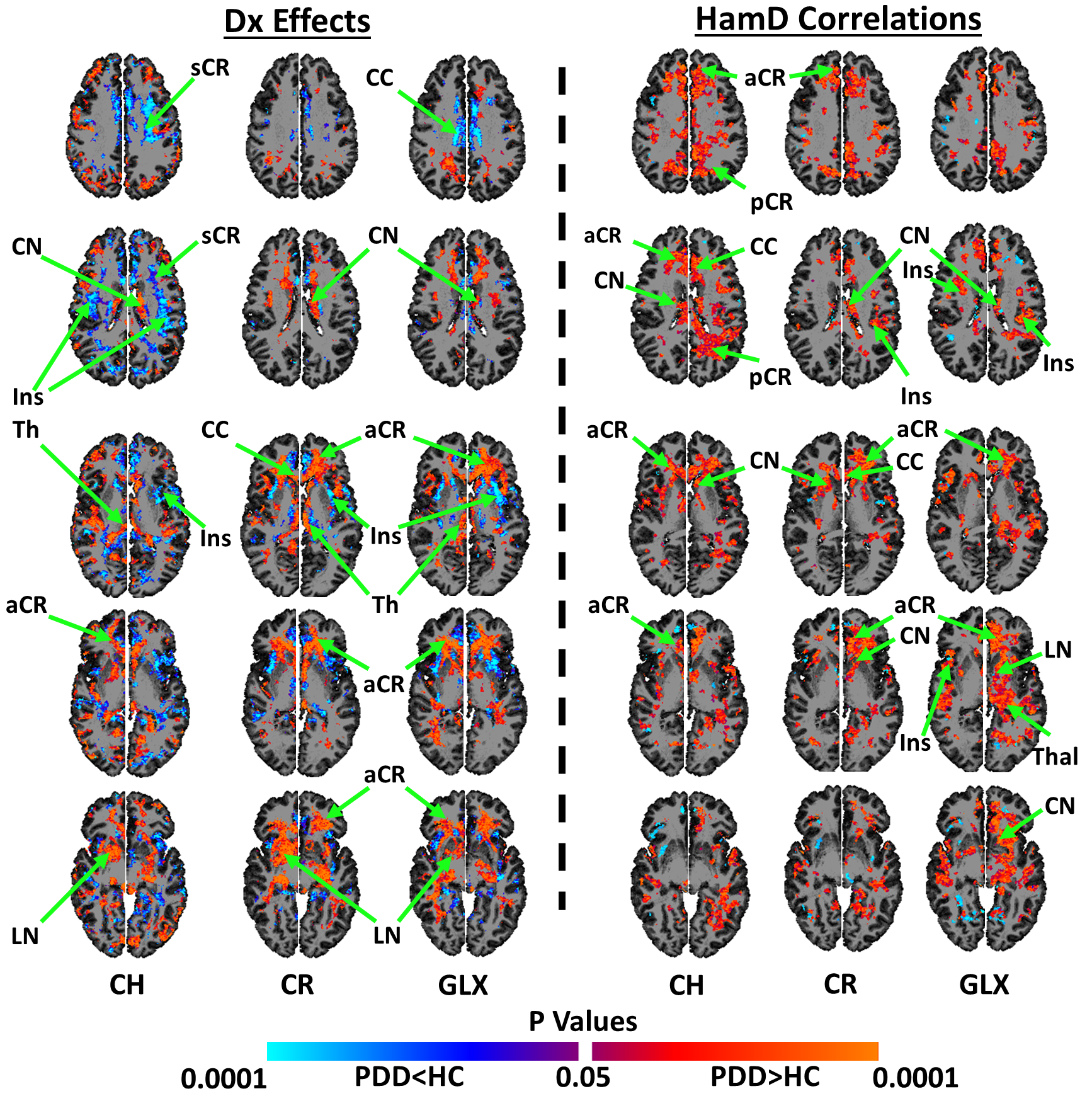

Supplement: S4 Fig — At baseline, we separately compared Ch, Cr, and Glx concentrations in 41 patients with dysthymic disorder (PDD) relative to 29 healthy controls (Dx Effects). We also correlated metabolite levels with symptom severity in the 41 patients (HamD Correlations). Dx Effects: Patients, relative to controls, had higher metabolite concentrations across large portions of the brain, especially in the caudate nucleus (CN), anterior corona radiata (aCR), thalamus (Thal), right lenticular nucleus (putamen and globus pallidus), and posterior corona radiata (pCR); and had lower concentration lower in the corpus callosum (CC), superior corona radiata(sCR), and insula (Ins). HamD Correlations: Symptom severity, measured using the Hamilton Depression Rating Scale (HDRS), correlated positively with all metabolite levels (i.e., patients with higher metabolite concentrations had more severe symptoms), especially in the sCR, CN, and pCR. Therefore, patients relative to controls had higher metabolite concentrations, especially in the inferior portions of the brain, and those with higher levels had more severe symptoms. Transverse brain slices are shown in the radiological orientation. We controlled for false positives in all analyses using a false discovery rate (FDR) procedure and covaried for age and sex. We subsequently applied a cluster threshold that suppressed all findings of spatial extent smaller than 100 contiguous voxels. P-values are color coded, with positive associations displayed in warm colors (orange and red) and inverse associations in cool colors (cyan and blue). (TIF) [file pone.0219679.s004.TIF]

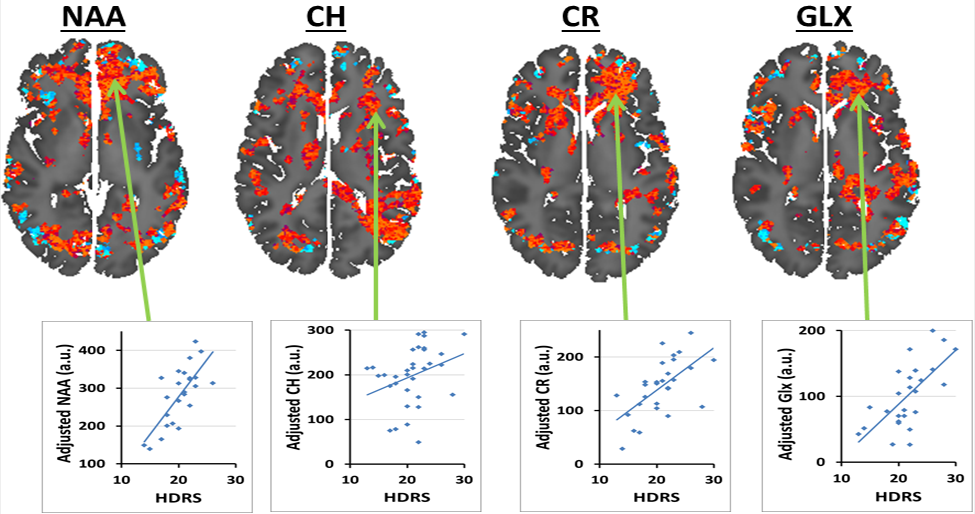

Supplement: S5 Fig — We generated scatterplots for visually assessing how metabolite concentrations were associated with symptom severity at a select region in the brain that survived FDR correction for multiple comparisons. Metabolite concentrations along the Y-axis are in arbitrary internal units normalized by the amount of background noise. Furthermore, metabolite concentrations are adjusted for differing age and sex across participants and corrected for partial volume effects from differing tissue composition within each MRS voxel. These plots show that positive associations of increasing metabolite concentrations with increasing symptom severity are not a consequence of outlying values in the data. (TIF) [file pone.0219679.s005.TIF]

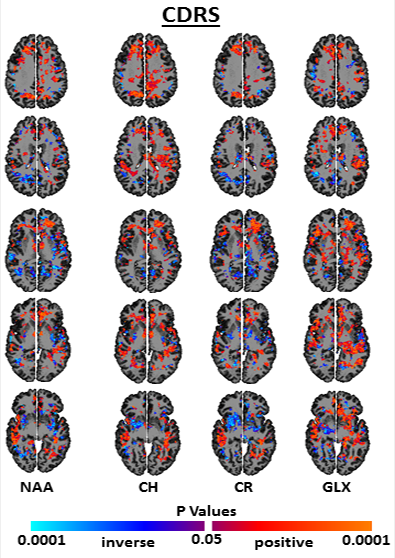

Supplement: S6 Fig — We assessed symptom severity in patients using both the 24-item Hamilton Depression Rating Scale (HDRS)[31] and the Cornell Dysthymia Rating scale (CDRS).[32] These maps show that metabolite concentrations were positively associated with symptom severity measured using CDRS across most regions of the brain. All maps were FDR-corrected at a false discovery rate of 0.05, covarying for age and sex. We subsequently applied a cluster threshold that suppressed all findings of spatial extent smaller than 100 contiguous voxels. (TIF) [file pone.0219679.s006.TIF]

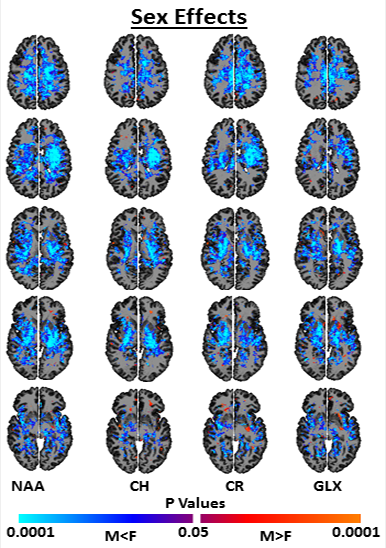

Supplement: S7 Fig — We assessed how NAA, Ch, Cr, and Glx concentrations in males differed from those in females at baseline using our entire cohort of 41 patients and 29 healthy controls while covarying for age and diagnosis. These analyses showed that males had significantly lower levels of metabolites across several regions of the brain. We controlled for false positives using a false discovery rate (FDR) procedure. We applied a cluster-level threshold of 100 voxel and color encoded P-values such that brain regions with higher metabolite concentrations in males are displayed in warm colors (orange and red) and those with lower concentrations in males are displayed in cool colors (cyan and blue). (TIF) [file pone.0219679.s007.TIF]

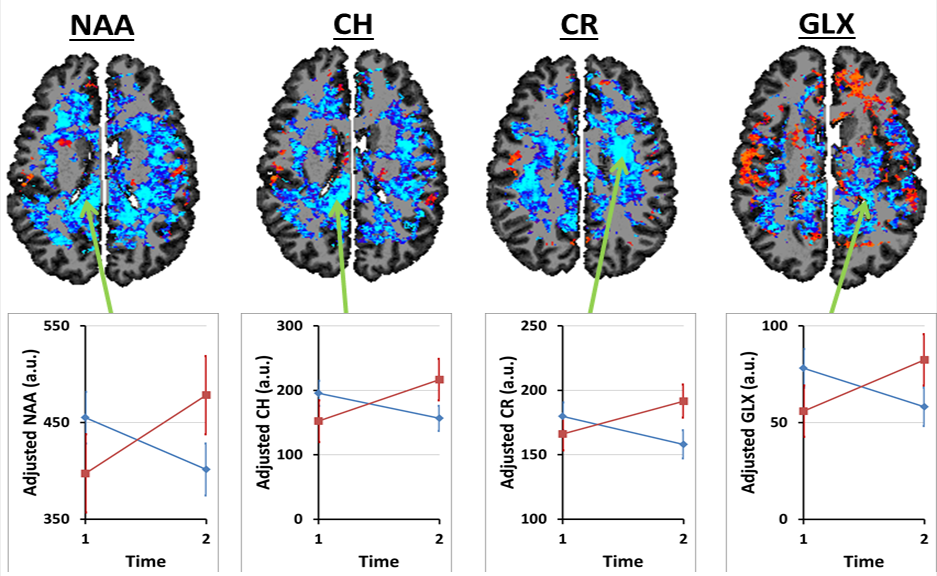

Supplement: S8 Fig — Over the 10-week period of the clinical trial, changes in metabolite concentrations in the duloxetine arm differed from those in the placebo arm of the trial (Fig 2). To understand better those changes, we generated scatterplots for changes metabolite concentrations at a brain region with significant effects of treatment-by-time interaction on metabolite concentrations. Metabolite concentrations along the Y-axis are in arbitrary internal units normalized by the amount of background noise. Furthermore, metabolite concentrations are adjusted for the age and sex of participants and are corrected for partial volume effects from differing tissue composition within each MRS voxel. These scatterplots show that at baseline (i.e., Time 1) concentrations in patients randomized to the duloxetine arm did not differ significantly from those in patients randomized to the placebo arm. However, concentrations increased in placebo-treated patients (brown) but declined in duloxetine-treated patients (blue) by the end of the trial (i.e., Time 2). (TIF) [file pone.0219679.s008.TIF]

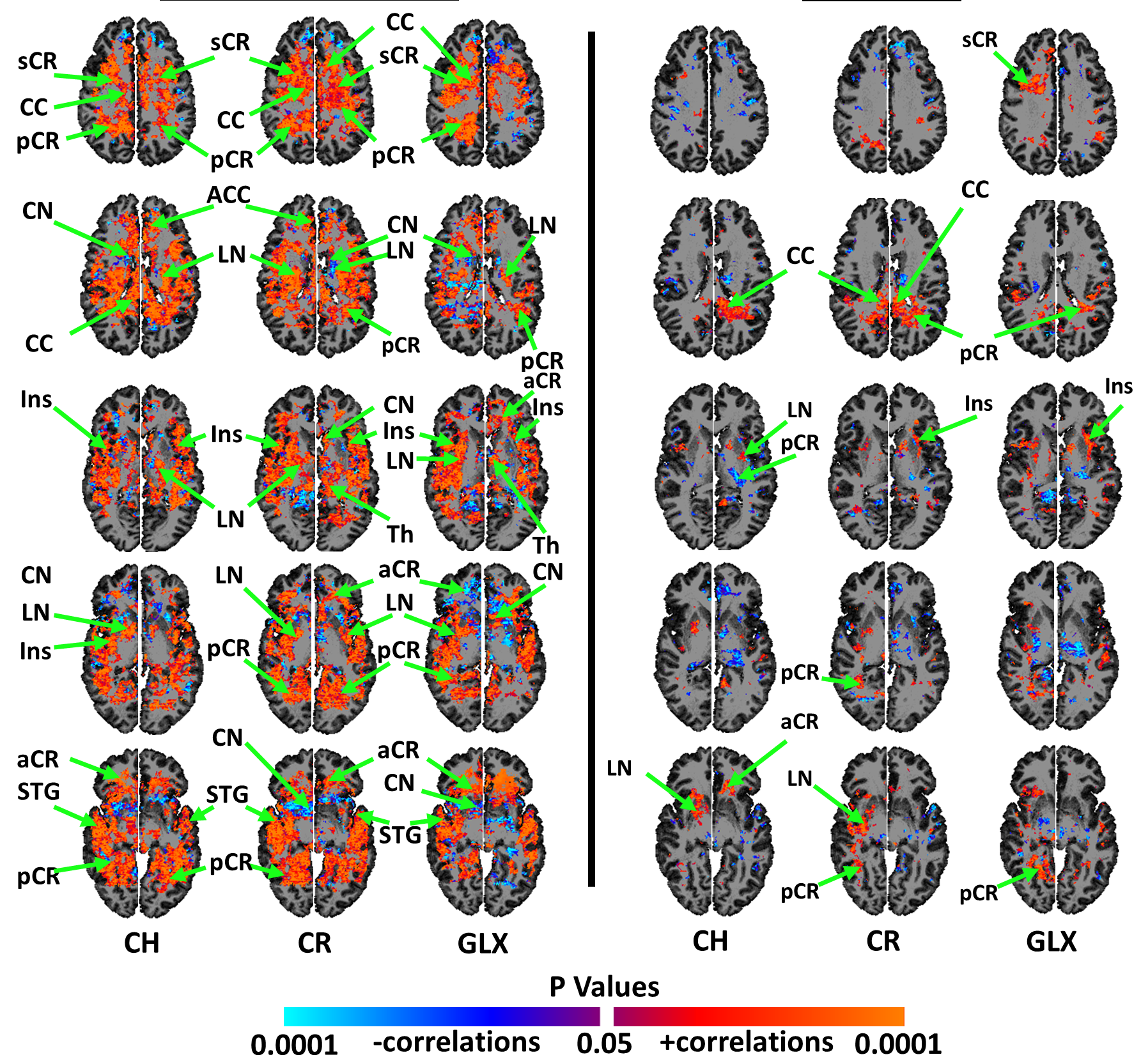

Supplement: S9 Fig — Left Panel: Using repeated measures analyses, we assessed within duloxetine-treated patients how concentrations of Ch, Cr, and Glx changed separately with change in their symptom severity. These analyses showed that in general the change in metabolite levels were positively associated with change in symptom severity: i.e., metabolite levels decreased towards healthy values as symptom severity decreased in duloxetine-treated patients. Right Panel: We subsequently applied longitudinal mediation analyses to assess whether changes in symptom severity mediated the treatment effects on changes in metabolite concentration. These analyses showed that symptom severity significantly mediated the change in Glx concentrations as a consequence of treatment in the caudate nucleus (CN). Mediation analyses provided no statistical evidence for the alternate hypothesis, that the change in metabolite concentrations mediated the treatment effects on change in symptom severity. All maps are FDR-corrected at p<0.05, and analyses included age and sex as covariates. We subsequently applied a cluster threshold that suppressed all findings of spatial extent smaller than 100 contiguous voxels. Positive associations were coded in warm colors (orange and red); inverse associations were coded in cool colors (cyan and blue). (TIF) [file pone.0219679.s009.TIF]

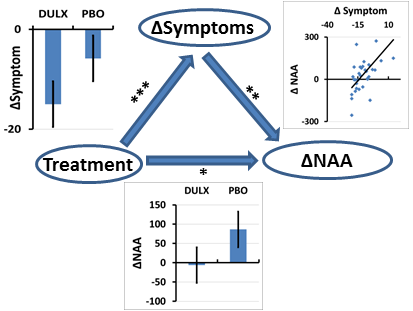

Supplement: S10 Fig — The scatter plot shows the associations among treatment, change in symptom severity, and change in the levels of NAA in the caudate nucleus, where the mediating effects of symptom severity were statistically significant. Treatment significantly decreased symptom severity in duloxetine-treated patients (p = 2.4 x 10^−4^). The decline in symptom severity correlated significantly with the decline in NAA concentrations (p = 0.014), and treatment correlated significantly with the decline in Glx levels (p = 0.048).* = P-value<0.05; ** = P-value<0.01; *** = P-value<0.001. (TIF) [file pone.0219679.s010.TIF]

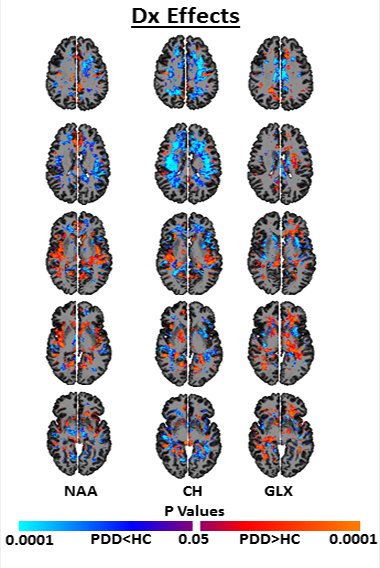

Supplement: S11 Fig — We assessed how baseline concentrations of NAA, Ch, and Glx in 41 patients differed from those in 29 healthy controls while covarying for age, sex, and Cr concentration. These analyses showed that metabolite concentrations in patients differed from those in healthy controls across the same brain regions and in the same direction as those in analyses without covarying for Cr (Fig 1, left panel). However, because Cr levels changed in the same direction as NAA and Glx levels and in the opposite direction as Ch levels, differences in NAA and Glx levels were attenuate whereas differences in Ch levels were accentuated. We controlled for false positives using a false discovery rate (FDR) procedure and covaried for age and sex. We subsequently applied a cluster threshold that suppressed all findings of spatial extent smaller than 100 contiguous voxels. P-values are color coded, with positive associations displayed in warm colors (orange and red) and inverse associations in cool colors (cyan and blue). (TIF) [file pone.0219679.s011.TIF]
