# Supplementary material for: Effects of the antidepressant medication duloxetine on brain metabolites in persistent depressive disorder: A randomized, controlled trial
Source: PLoS One. 2019 Jul 19;14(7):e0219679. doi: 10.1371/journal.pone.0219679 (PMC6641507; doi:10.1371/journal.pone.0219679)
Supplement: S1 File — (DOCX) [file pone.0219679.s012.docx]

# Supporting Material

# Supporting Methods

**Study Design** We conducted a traditional RCT with *a priori* ascertained, fixed number of participants and conducted all data processing and statistical analyses after completion of the trial. We acquired MRS data at baseline and at the end of the trial in 41 patients and once in 29 healthy controls age- and sex-matched to patients (**S1 Fig**).

**Abbreviations** We use the following abbreviations throughout the text and figures. **RCT**, Randomized, controlled trial; **PDD**, persistent depressive disorder; **DULX**, Duloxetine; **PBO**, placebo; **HC**, healthy controls; **R**, Right Hemisphere; **L,** Left Hemisphere; **CDRS**, Cornell Dysthymia Rating scale; **NAA**, N-acetyl Aspartate; **Ch**, Choline; **Cr**, Creatine+Phosphocreatine; **Glx**, Glutamate+Glutamine; **ppm**, parts per million; **CC**, corpus callosum; **sCR**, superior corona radiata; **CN**, caudate nucleus; **aCR**, anterior corona radiata; **Ins**, insular, **Th**, thalamus; **LN**, lenticular nucleus (putamen and globus pallidus); **pCR**, posterior corona radiata; **a.u.**, arbitrary units; **M**, Male; **F**, Female; **FDR**, false discovery rate.

**MRI Processing**

All MRI data were processed blind to the order of data acquisition, participant characteristics, and patient assignments to the treatment arms.

Anatomical MRI We applied automated tools to correct for large-scale variations in image intensities[[2](#_ENREF_2)] and to remove extracerebral tissue.[[3](#_ENREF_3)] We then removed connecting dura manually on each slice in the sagittal, axial, and coronal views. We first coregistered all participant brains to an initial template using a similarity transform that maximized mutual information[[5](#_ENREF_5)] and then applied a high-dimensional, nonrigid fluid-flow algorithm[[6](#_ENREF_6)] to warp each brain to the exact size and shape of the template brain. Brain tissue was segmented as gray or white matter using a semi-automated method where an expert neuroanatomist sampled gray-scale values of both cortical gray and white matter at 4 standard locations throughout the brain. We then averaged these values and used them as a threshold to generate initial tissue definitions that we then edited to remove subcortical gray matter. The test-retest intraclass correlation coefficient (ICC)[[7](#_ENREF_7)] for defining the cortex was >0.98.

**Supporting Statistical Analyses**

We excluded from further statistical analysis data at MRS voxels with poor quality spectrum due to either contamination with the lipid signal, suppression of signal by saturation bands, or line broadening cause by magnetic field inhomogeneities or insufficient shimming of the static magnetic field. We identified and excluded these voxels by carefully examining the spectra at each voxel for every participant. Most of these problematic voxels were along the periphery of the brain, within cortical gray matter. The MRS data in white matter and subcortical brain regions, however, were of excellent quality in all participants, leading to accurate spectral fitting for quantifying metabolite concentrations **(S1 Fig)**. Because data from each participant were processed separately from other participants, voxels around brain periphery of the brain had acceptable MRS data from differing numbers of participants at each voxel in the template space. We, therefore, performed statistical analyses only in voxels where we had valid MRS data in at least half the patients and half the healthy controls (**S2 Fig**).

# Supporting Results

**Baseline Assessments** The voxelwise maps of average metabolite concentrations show that although metabolite concentrations were similar, patients relative to healthy controls had lower concentration of CH and higher concentrations of NAA, CR, and Glx across most of the brain regions (**S3 Fig**).

Baseline concentration of CH, Cr, and GLX were increased in aCR, posterior corona radiata (pCR), CN, LN, and Th, but lower in WM of the corpus callosum (CC) and superior corona radiata (sCR) and in GM region insula (Ins). (**S4 Fig, left panel**). Furthermore, metabolite concentrations within patients were positively associated with symptom severity (**S4 Fig, right panel**). We subsequently assessed whether metabolite concentrations were correlated and whether those correlations differed by group. We applied a general linear model (GLM) $Met_{1}=\beta_{0}+\beta_{1}*Age+\beta_{2}*sex+\beta_{3}*Met_{2}+\beta_{4}*Dx+\beta_{5}*Dx*Met_{2}$, while controlling for age and sex, $Met_{1}$ is the concentration of one metabolite, $Met_{2}$ is the concentration of another metabolite, and $Dx$ is diagnosis. We assessed significant correlations between metabolite concentrations by testing whether $\beta_{3}$ was significantly differs from zero, and then assessed whether that correlation differs by group by testing whether $\beta_{5}$ was significantly differs from zero. Conducting these GLMs for various combinations for $Met_{1}$ and $Met_{2}$ showed that concentrations of metabolites were significantly intercorrelated, and those correlations did not differ significantly by diagnosis.

To understand these positive associations, we generated scatterplots for each metabolite at a selected region in the brain (**S5 Fig**). These plots show that positive associations are not a consequence of outlier values in metabolite concentration.

Furthermore, baseline metabolite concentrations were positively associated with symptom severity measured using either the HDRS (**Fig 3**, ***right panel* & S4 Fig, right panel**) or the CDRS (**S6 Fig**) across most of the brain regions.

Finally, males relative to females had lower baseline concentrations across large portions of the brain (**S7 Fig**). In our trial, although patients were randomly assigned to one of the two treatment arms, the number of males was significantly higher in the placebo arm than in the duloxetine arm. However, baseline metabolite concentrations in patients assigned to the duloxetine arm did not differ from those in the placebo arm. Furthermore, at the end of the trial, placebo-treated patients relative to duloxetine-treated patients had higher metabolite concentrations even though the placebo arm had higher number of males that the duloxetine arm. Therefore, differing number of males in the two treatment arms did not confound the findings reported in this study

**Change during the RCT** Metabolite levels changed differentially between the duloxetine- and placebo-treated patients during the trial (**Fig 4**). At the start of the trial, patients relative to healthy controls had higher metabolite concentrations through out most of the brain (**Fig 3, left panel & S4 Fig, left panel**). By the end of trial, metabolite levels in duloxetine-treated patients were statistically indistinguishable from control values in many regions where metabolite concentrations were elevated at baseline (in WM regions of CCb, SCR, EC, IFOF, PTR, and in GM regions of SFG, CN, Th, SFG, ACC, Ins), but did not entirely normalize in the internal capsule and SLF (**S8 Fig**). In contrast, metabolite levels in placebo-treated patients remained significantly higher across most of the brain, including WM regions of CC, sCR, aCR, pCR, and GM regions of CN, LN, Th, Ins (**S8 Fig**).

**Mediation** **Analyses** Scatter plots for mediation analyses show that symptom severity significantly mediated the treatment effects on metabolite concentration within the caudate nucleus, and that these mediations are not because of outlying values (**S9 & S10 Figs**).

**Supporting Discussion**

**Normalizing Metabolite Concentrations to Noise**

Unlike previous MRS studies that assessed ratio of metabolite concentrations relative to Cr, we assessed how metabolite values normalized to the background noise in each MRS spectrum. We used metabolite concentrations because we were primarily interested in whether the patients relative to healthy had abnormal metabolite concentrations and whether treatment with the active medication normalized those levels. These hypotheses, in the RCT design that we employed, are most validly tested in a repeated measures analysis where the change in metabolite levels are compared against baseline values, and those changes are then compared across treatment arms. Our analyses showed that abnormally high baseline concentrations normalized and that all concentrations changed similarly over the 10-week trial. Using a ratio measure when concentrations change similarly may lead to invalid and erroneous inferences, or at least to findings that would be inscrutable to interpretation, because both the numerator and denominator could change over time as well as differentially across treatment arms. If we were to report ratio measurements, then those findings would need to be deconstructed into absolute metabolite measures separately for both the numerator and denominator anyway, to have any hope of understanding what changes were truly driving the findings. Perhaps certain procedures could mitigate to some extent this confusion that accompanies the use of ratios, such as using Cr concentration at either baseline, end of the trial, or using the average of the two values. However, these are *ad hoc* procedures, which may lead to conflicting findings. Our use of metabolite concentrations that are normalized to noise values overcomes these difficulties, thereby leading to a clearer understanding of how metabolites concentrations separately change during the clinical trial.

Nevertheless, we also assessed how baseline concentrations in patients differed from healthy controls while including voxel-wise Cr concentrations as a covariate: covariation is statistically preferable than using ratio of two random variables for reducing the effects of noise (**S11 Fig**). When covarying for Cr, these baseline analyses showed that metabolite concentrations in patients differed from those in healthy controls across the same brain regions and in the same direction as in analyses without covarying for Cr (**Fig 3**, *left panel* & **S4 Fig**). However, because Cr levels changed in the same direction as NAA and Glx levels and in the opposite direction as Ch levels, group differences in NAA and Glx levels were attenuated, whereas differences in Ch levels were accentuated when using Cr as a covariate. In other words, differences in metabolite concentrations when we covaried for Cr were in general similar to those when we did not covary for it. However, those differences were modified depending upon how Cr concentrations were associated with concentrations of other metabolite, leading to confusion in understanding whether and by how much metabolite levels were abnormal in patients relative to healthy controls. We therefore present findings in this study using metabolite levels normalized to the background noise in the MRS spectrum.

**Possible Cellular and Physiological Determinants**

Except for Ch levels in superior WM, all metabolite levels were affected similarly by the illness and its treatment. The similar patterns of treatment-induced change across metabolites could be a consequence of underlying baseline alterations and medication-induced alterations of one or more physiological processes, including neuroplastic structural changes, the bioenergetic consequences of depressive symptoms, mitochondrial dysfunction, glial cell dysfunction, or neuroinflammatory changes associated with illness-induced stress (Supporting Material). These physiological processes are not independent of one another, and therefore dysfunction in one may cause dysfunction in another. For example, microglial cells activated by neuroinflammation release nitric oxide and reactive oxygen species, leading to nitrosative and oxidative damage to mitochondrial DNA and impairment of mitochondrial functioning.[[8](#_ENREF_8)] Conversely, mitochondrial dysfunction has been hypothesized to activate microglia, generating a vicious cycle that culminates in neuronal death.[[9](#_ENREF_9)] Thus, each of these physiological processes, either individually or in concert, may be responsible for the similar findings observed across all metabolites at baseline and during duloxetine treatment.

Neural Plasticity We previously provided in this same cohort of patients compelling evidence for the presence of an adaptive neuroplasticity in the cerebral cortex that functions to reduce the severity of depressive symptoms. At baseline patients had a thicker cortical mantle across large portions of their cerebral surface compared with controls, and in inverse proportion to the severity of their depressive symptoms, suggesting that the thickening was helpful and compensatory in nature. Medication normalized thickness values, whereas placebo had no effect. Similar to the our present findings, mediation analyses showed that normalization of cortical thickness did not produce symptom change, but instead was itself a consequence of the medication-induced improvement of symptoms, presumably because neuroplastic compensation was no longer needed.[[10](#_ENREF_10)] Plastic changes in the cortical gray matter likely represented an increase in dendritic arborization and synaptic density, which would increase metabolic demands and produce the elevated metabolite concentrations we observed in patients at baseline.[[11-13](#_ENREF_11)] By the end of the 10-week trial, cortical thickness had normalized in duloxetine-treated patients but not in those treated with placebo; normalization of cortical thickness would likewise have normalized metabolism and metabolite levels in those treated with duloxetine but not placebo, consistent with our observations in the present study. Cortical thickness correlated inversely with the symptom severity, however, and if neural plasticity was driving elevated metabolite concentrations, then those concentrations should also have correlated inversely with symptom severity, which is opposite the positive correlations we observed. These opposing correlations of symptom severity with cortical thickness measures and metabolite concentrations suggest that the elevated metabolite concentrations are likely not a simple byproduct of compensatory neuroplastic changes in PDD.

Bioenergetics NAA, Cr, Ch, and Glx participate in key metabolic processes in the brain, and any processes that alter brain energetics likely will alter all metabolite levels similarly. In particular, higher levels of the excitatory neurotransmitters glutamate likely represent more neural firing, which will in turn drive up metabolic activity. In that case, higher Cr levels, a short-term ATP buffer,[[14](#_ENREF_14)]^-55^ could indicate a greater need for short-term energy storage to support greater excitatory transmission in patients relative to controls. It could also increase NAA levels to aid long-term energy storage, given that NAA is synthesized in mitochondria[[15-17](#_ENREF_15)] from the glycolysis end product, acetyl-CoA, and is conjectured to serve as a storage substrate for longer-term energy expenditure,[[18](#_ENREF_18), [19](#_ENREF_19)] and indeed NAA levels in general correlate with the rate of brain glucose metabolism.[[20](#_ENREF_20), [21](#_ENREF_21)] Normalization of Glx following duloxetine treatment likely would have reduced regional metabolic demands, which in turn would have contributed to normalization of both short- and long-term energy storage in the form of Cr and NAA, respectively. Finally, low WM levels of Ch, a marker for cellular membrane turnover,[[22](#_ENREF_22), [23](#_ENREF_23)] could indicate lower WM myelin content, as has been reported in prefrontal and limbic regions by *post mortem* studies[[24-27](#_ENREF_24)] of MDD; they could also indicate, however, that its carbon substrate is being diverted from the construction of cell membranes to meet cell-energetic demands.

Mitochondrial Dysfunction Mitochondria are cell organelles that generate most of the cell’s energy. Their dysfunction can alter levels of several metabolites similarly.[[28](#_ENREF_28), [29](#_ENREF_29)] Disrupted mitochondrial functioning has been implicated in affective disorders[[28](#_ENREF_28), [30](#_ENREF_30), [31](#_ENREF_31)] and has been reported to normalize with antidepressant medications.[[32](#_ENREF_32)] Mitochondrial dysfunction, however, would seem more likely to yield abnormally low metabolism and therefore generate reduced metabolite concentrations relative to control values, not the elevated concentrations that we observed. Substantial mitochondrial dysfunction from any cause would be expected to generate brain lactate, especially in the presence of the increased metabolic demand that seems to have been present in our PDD patients. Testing for lactate in MRS data from our participants is possible[[33](#_ENREF_33)] and will be a future effort.

Glial Cell Dysfunction Glial cells metabolize glucose[[34](#_ENREF_34)] and contribute importantly to the brain’s overall metabolic demands.[[35](#_ENREF_35), [36](#_ENREF_36)] Altered glial cell numbers or functioning could account in part for the elevated concentrations across all metabolites we measured, particularly in WM.[[37](#_ENREF_37)] Astrocytes, the most numerous glial cells, build and maintain the blood brain barrier,[[38](#_ENREF_38), [39](#_ENREF_39)] regulate blood flow,[[40](#_ENREF_40)] modulate extracellular levels of neurotransmitters glutamate,[[41](#_ENREF_41)] GABA,[[42](#_ENREF_42)] serotonin,[[43](#_ENREF_43)] and dopamine,[[44](#_ENREF_44)] and are vital participants in synaptic functioning.[[45](#_ENREF_45)] They play a particularly important role in the homeostasis of glutamate concentration, as they take up glutamate at the synaptic cleft, metabolize it to glutamine,[[46](#_ENREF_46)] and release it back into the extracellular space for uptake by glutamatergic neurons. The loss or degeneration of astrocytes[[47](#_ENREF_47), [48](#_ENREF_48)] reported in prefrontal[[49](#_ENREF_49)] and limbic cortex[[50](#_ENREF_50)] in depressed patients could reduce glutamate metabolism[[51](#_ENREF_51)] and increase glutamate concentrations,[[37](#_ENREF_37)] consistent with our findings in PDD. Oligodendrocytes, smaller and less abundant than astrocytes, provide trophic support to neurons in GM and ensheath axons with myelin in WM.[[37](#_ENREF_37)] A reduced density or functioning of oligodendrocytes[[52](#_ENREF_52), [53](#_ENREF_53)] has been linked to disrupted mood regulation.[[54](#_ENREF_54)] Because oligodendrocytes catabolize[[55](#_ENREF_55), [56](#_ENREF_56)] NAA synthesized within neurons,[[57](#_ENREF_57), [58](#_ENREF_58)] their lower numbers could reduce catabolism and in turn increase NAA concentration in PDD. Finally, altered microglia, the brain analogues of macrophages in the periphery, may initiate or sustain inflammation, which in turn would alter brain metabolism.[[49](#_ENREF_49)]

Inflammation Prior studies[[59-61](#_ENREF_59)] have implicated neuroinflammation in the pathogenesis of MDD,[[62-66](#_ENREF_62)] possibly as a consequence of the release of proinflammatory cytokines[[67](#_ENREF_67)] in response to stress.[[61](#_ENREF_61), [68](#_ENREF_68)] Cytokines target and activate astrocytes, oligodendrocytes, and microglia,[[69](#_ENREF_69)] increasing their number and size[[70](#_ENREF_70), [71](#_ENREF_71)] as a proinflammatory response to aid recovery from stress and injury. Activated astrocytes and oligodendrocytes release neurotrophic factors that enhance neuronal survival and growth, synaptic plasticity, synaptic efficiency, and axonal repair processes.[[72](#_ENREF_72)] Activation of glial cells increases brain metabolism and can thereby increase multiple brain metabolites simultaneously[[73](#_ENREF_73)] and could account for the similar concentration changes we observed across all metabolites.

# References

1. Hamilton M. A rating scale for depression. J Neurol Neurosurg Psychiatry. 1960;23:56-62. Epub 1960/02/01. PubMed PMID: 14399272; PubMed Central PMCID: PMC495331.

2. Sled GJ, Zijdenbos AP, Evans AC. A Nonparametric Method for Automatic Correction of Intensity Nonuniformity in MRI Data. IEEE Trans of Medical Imaging. 1998;17(1):87-97.

3. Shattuck DW, Leahy RM. BrainSuite: An Automated Cortical Surface Identification Tool. Medical Image Analysis. 2002;8(2):129-42.

4. Hellerstein DJ, Batchelder ST, Lee A, Borisovskaya M. Rating dysthymia: an assessment of the construct and content validity of the Cornell Dysthymia Rating Scale. J Affect Disorders. 2002;71(1-3):85-96. doi: Pii S0165-0327(01)00371-8

Doi 10.1016/S0165-0327(01)00371-8. PubMed PMID: ISI:000177824700010.

5. Viola P, Wells, W. M., editor Alignment by Maximization of Mutual Information. IEEE Proc of the 5th Int Conf on Computer Vision; 1995 June 20-23; Boston, MA.

6. Christensen GE, Joshi SC, Miller MI. Volumetric Transformation of Brain Anatomy. IEEE Transactions on Medical Imaging. 1997;16(6):1369-83.

7. Shrout PE, Fleiss JL. Intraclass correlations: uses in assessing rater reliability. Psychol Bull. 1979;86:420-8.

8. Lu FM, Selak M, O'Connor J, Croul S, Lorenzana C, Butunoi C, et al. Oxidative damage to mitochondrial DNA and activity of mitochondrial enzymes in chronic active lesions of multiple sclerosis. J Neurol Sci. 2000;177(2):95-103. doi: Doi 10.1016/S0022-510x(00)00343-9. PubMed PMID: ISI:000089507100002.

9. Di Filippo M, Chiasserini D, Tozzi A, Picconi B, Calabresi P. Mitochondria and the Link Between Neuroinflammation and Neurodegeneration. Journal of Alzheimers Disease. 2010;20:S369-S79. doi: 10.3233/Jad-2010-100543. PubMed PMID: ISI:000280479600010.

10. Bansal R, Hellerstein DJ, Peterson BS. Evidence for Neuroplastic Compensation in the Cerebral Cortex of Persons with Dysthymia. Mol Psychiatr. 2017;In Press.

11. Hyder F, Rothman DL, Bennett MR. Cortical energy demands of signaling and nonsignaling components in brain are conserved across mammalian species and activity levels. Proc Natl Acad Sci U S A. 2013;110(9):3549-54. Epub 2013/01/16. doi: 10.1073/pnas.1214912110. PubMed PMID: 23319606; PubMed Central PMCID: PMC3587194.

12. Potter RL. Regional differences of respiration in the bullfrog brain based on cell density. J Neurobiol. 1977;8(2):133-9. Epub 1977/03/01. doi: 10.1002/neu.480080205. PubMed PMID: 300787.

13. Magistretti PJ, Allaman I. A Cellular Perspective on Brain Energy Metabolism and Functional Imaging. Neuron. 2015;86(4):883-901. doi: 10.1016/j.neuron.2015.03.035. PubMed PMID: ISI:000354878400006.

14. Bessman SP, Geiger PJ. Transport of Energy in Muscle - the Phosphorylcreatine Shuttle. Science. 1981;211(4481):448-52. doi: DOI 10.1126/science.6450446. PubMed PMID: ISI:A1981KZ36500008.

15. Ledeen RW. Lipid-Metabolizing Enzymes of Myelin and Their Relation to the Axon. Journal of Lipid Research. 1984;25(13):1548-54. PubMed PMID: ISI:A1984ABM4700018.

16. Madhavarao CN, Chinopoulos C, Chandrasekaran K, Namboodiri MAA. Characterization of the N-acetylaspartate biosynthetic enzyme from rat brain. J Neurochem. 2003;86(4):824-35. doi: 10.1046/j.1471-4159.2003.01905.x. PubMed PMID: ISI:000184451400005.

17. Wiame E, Tyteca D, Pierrot N, Collard F, Amyere M, Noel G, et al. Molecular identification of aspartate N-acetyltransferase and its mutation in hypoacetylaspartia. Biochem J. 2010;425:127-36. doi: 10.1042/Bj20091024. PubMed PMID: ISI:000273585000013.

18. Ariyannur PS, Moffett JR, Manickam P, Pattabiraman N, Arun P, Nitta A, et al. Methamphetamine-induced neuronal protein NAT8L is the NAA biosynthetic enzyme: Implications for specialized acetyl coenzyme A metabolism in the CNS. Brain Research. 2010;1335:1-13. doi: 10.1016/j.brainres.2010.04.008. PubMed PMID: ISI:000278789400001.

19. Francis JS, Strande L, Markov V, Leone P. Aspartoacylase supports oxidative energy metabolism during myelination. J Cerebr Blood F Met. 2012;32(9):1725-36. doi: 10.1038/jcbfm.2012.66. PubMed PMID: ISI:000308390900007.

20. O'Neill J, Eberling IL, Schuff N, Jagust W, Reed B, Soto G, et al. Method to correlate H-1 MRSI and (18)FDG-PET. Magnetic Resonance in Medicine. 2000;43(2):244-50. doi: Doi 10.1002/(Sici)1522-2594(200002)43:2<244::Aid-Mrm11>3.0.Co;2-2. PubMed PMID: ISI:000084993500011.

21. Moreno A, Ross BD, Bluml S. Direct determination of the N-acetyl-L-aspartate synthesis rate in the human brain by C-13 MRS and [1-C-13]glucose infusion. J Neurochem. 2001;77(1):347-50. doi: DOI 10.1046/j.1471-4159.2001.00282.x. PubMed PMID: ISI:000167835200035.

22. Klein J, Gonzalez R, Koppen A, Loffelholz K. Free Choline and Choline Metabolites in Rat-Brain and Body-Fluids - Sensitive Determination and Implications for Choline Supply to the Brain. Neurochemistry International. 1993;22(3):293-300. doi: Doi 10.1016/0197-0186(93)90058-D. PubMed PMID: ISI:A1993KN73600010.

23. Poptani H, Gupta RK, Gupta K, Roy R, Pandey R, Jain VK, et al. Characterization of Intracranial Mass Lesions with in-Vivo Proton Mr Spectroscopy. American Journal of Neuroradiology. 1995;16(8):1593-603. PubMed PMID: ISI:A1995RV03300007.

24. Tham MW, Woon PS, Sum MY, Lee TS, Sim K. White matter abnormalities in major depression: evidence from post-mortem, neuroimaging and genetic studies. J Affect Disord. 2011;132(1-2):26-36. doi: 10.1016/j.jad.2010.09.013. PubMed PMID: 20889213.

25. Liu J, Dietz K, DeLoyht JM, Pedre X, Kelkar D, Kaur J, et al. Impaired adult myelination in the prefrontal cortex of socially isolated mice. Nat Neurosci. 2012;15(12):1621-3. Epub 2012/11/13. doi: 10.1038/nn.3263. PubMed PMID: 23143512; PubMed Central PMCID: PMC3729624.

26. Fuster JM. Frontal lobe and cognitive development. J Neurocytol. 2002;31(3-5):373-85. Epub 2003/06/20. PubMed PMID: 12815254.

27. Fox NA, Bell MA. Electrophysiological indices of frontal lobe development. Relations to cognitive and affective behavior in human infants over the first year of life. Annals of the New York Academy of Sciences. 1990;608:677-98; discussion 98-704. Epub 1990/01/01. PubMed PMID: 2075966.

28. Stork C, Renshaw PF. Mitochondrial dysfunction in bipolar disorder: evidence from magnetic resonance spectroscopy research. Mol Psychiatry. 2005;10(10):900-19. Epub 2005/07/20. doi: 10.1038/sj.mp.4001711. PubMed PMID: 16027739.

29. Jou SH, Chiu NY, Liu CS. Mitochondrial dysfunction and psychiatric disorders. Chang Gung Med J. 2009;32(4):370-9. Epub 2009/08/12. PubMed PMID: 19664343.

30. Klinedinst NJ, Regenold WT. A mitochondrial bioenergetic basis of depression. J Bioenerg Biomembr. 2015;47(1-2):155-71. Epub 2014/09/30. doi: 10.1007/s10863-014-9584-6. PubMed PMID: 25262287.

31. Gardner A, Boles RG. Beyond the serotonin hypothesis: mitochondria, inflammation and neurodegeneration in major depression and affective spectrum disorders. Prog Neuropsychopharmacol Biol Psychiatry. 2011;35(3):730-43. Epub 2010/08/10. doi: 10.1016/j.pnpbp.2010.07.030. PubMed PMID: 20691744.

32. Adzic M, Brkic Z, Bulajic S, Mitic M, Radojcic MB. Antidepressant Action on Mitochondrial Dysfunction in Psychiatric Disorders. Drug Dev Res. 2016;77(7):400-6. Epub 2016/08/20. doi: 10.1002/ddr.21332. PubMed PMID: 27539538.

33. Goh S, Dong ZC, Zhang YD, DiMauro S, Peterson BS. Mitochondrial Dysfunction as a Neurobiological Subtype of Autism Spectrum Disorder Evidence From Brain Imaging. Jama Psychiat. 2014;71(6):665-71. doi: 10.1001/jamapsychiatry.2014.179. PubMed PMID: ISI:000336915600011.

34. Raichle ME. Cognitive neuroscience - Bold insights. Nature. 2001;412(6843):128-30. doi: Doi 10.1038/35084300. PubMed PMID: ISI:000169778700027.

35. Volkenhoff A, Weiler A, Letzel M, Stehling M, Klambt C, Schirmeier S. Glial Glycolysis Is Essential for Neuronal Survival in Drosophila. Cell Metabolism. 2015;22(3):437-47. doi: 10.1016/j.cmet.2015.07.006. PubMed PMID: ISI:000360453900016.

36. Trevisiol A, Nave KA. Brain Energy Metabolism: Conserved Functions of Glycolytic Glial Cells. Cell Metabolism. 2015;22(3):361-3. doi: 10.1016/j.cmet.2015.08.010. PubMed PMID: ISI:000360453900007.

37. Smialowska M, Szewczyk B, Wozniak M, Wawrzak-Wlecial A, Domin H. Glial degeneration as a model of depression. Pharmacol Rep. 2013;65(6):1572-9. PubMed PMID: ISI:000330949400015.

38. Haines DE. Fundamental neuroscience. 3rd ed. Philadelphia: Churchill Livingstone; 2006. 567 p. p.

39. Kettenmann H, Kettenmann H, Ransom BR. Neuroglia. 3rd ed. Oxford ; New York: Oxford University Press; 2013. xxii, 930 p. p.

40. Zonta M, Angulo MC, Gobbo S, Rosengarten B, Hossmann KA, Pozzan T, et al. Neuron-to-astrocyte signaling is central to the dynamic control of brain microcirculation. Nature Neuroscience. 2003;6(1):43-50. doi: 10.1038/nn980. PubMed PMID: ISI:000180089300014.

41. Bezzi P, Gundersen V, Galbete JL, Seifert G, Steinhauser C, Pilati E, et al. Astrocytes contain a vesicular compartment that is competent for regulated exocytosis of glutamate. Nature Neuroscience. 2004;7(6):613-20. doi: 10.1038/nn1246. PubMed PMID: ISI:000221640300015.

42. Minelli A, DeBiasi S, Brecha NC, Zuccarello LV, Conti F. GAT-3, a high-affinity GABA plasma membrane transporter, is localized to astrocytic processes, and it is not confined to the vicinity of GABAergic synapses in the cerebral cortex. Journal of Neuroscience. 1996;16(19):6255-64. PubMed PMID: ISI:A1996VH85800035.

43. Hirst WD, Price GW, Rattray M, Wilkin GP. Serotonin transporters in adult rat brain astrocytes revealed by [H-3]5-HT uptake into glial plasmalemmal vesicles. Neurochemistry International. 1998;33(1):11-22. doi: Doi 10.1016/S0197-0186(05)80003-8. PubMed PMID: ISI:000074835500002.

44. Takeda H, Inazu M, Matsumiya T. Astroglial dopamine transport is mediated by norepinephrine transporter. N-S Arch Pharmacol. 2002;366(6):620-3. doi: 10.1007/s00210-002-0640-0. PubMed PMID: ISI:000179780500016.

45. Volterra A, Magistretti PJ, Haydon PG. The Tripartite Synapse: Glia in Synaptic Transmission. New York: Oxford University Press; 2002.

46. Waagepetersen HS, Sonnewald U, Schousboe A. Compartmentation of glutamine, glutamate, and GABA metabolism in neurons and astrocytes: Functional implications. Neuroscientist. 2003;9(5):398-403. doi: 10.1177/1073858403254006. PubMed PMID: ISI:000185615000020.

47. Miguel-Hidalgo JJ, Baucom C, Dilley G, Overholser JC, Meltzer HY, Stockmeier CA, et al. Glial fibrillary acidic protein immunoreactivity in the prefrontal cortex distinguishes younger from older adults in major depressive disorder. Biological Psychiatry. 2000;48(8):861-73. doi: Doi 10.1016/S0006-3223(00)00999-9. PubMed PMID: ISI:000165056200015.

48. Si XH, Miguel-Hidalgo JJ, O'Dwyer G, Stockmeier CA, Rajkowska G. Age-dependent reductions in the level of glial fibrillary acidic protein in the prefrontal cortex in major depression. Neuropsychopharmacol. 2004;29(11):2088-96. doi: 10.1038/sj.npp.1300525. PubMed PMID: ISI:000224806100016.

49. Rajkowska G, Miguel-Hidalgo JJ. Gliogenesis and glial pathology in depression. CNS Neurol Disord Drug Targets. 2007;6(3):219-33. Epub 2007/05/22. PubMed PMID: 17511618; PubMed Central PMCID: PMC2918806.

50. Altshuler LL, Abulseoud OA, Foland-Ross L, Bartzokis G, Chang S, Mintz J, et al. Amygdala astrocyte reduction in subjects with major depressive disorder but not bipolar disorder. Bipolar Disord. 2010;12(5):541-9. Epub 2010/08/18. doi: 10.1111/j.1399-5618.2010.00838.x. PubMed PMID: 20712756.

51. Hashimoto K, Sawa A, Iyo M. Increased levels of glutamate in brains from patients with mood disorders. Biol Psychiatry. 2007;62(11):1310-6. Epub 2007/06/19. doi: 10.1016/j.biopsych.2007.03.017. PubMed PMID: 17574216.

52. Hamidi M, Drevets WC, Price JL. Glial reduction in amygdala in major depressive disorder is due to oligodendrocytes. Biological Psychiatry. 2004;55(6):563-9. doi: 10.1016/j.biopsych.2003.11.006. PubMed PMID: ISI:000220109100002.

53. Uranova NA, Vostrikov VM, Orlovskaya DD, Rachmanova VI. Oligodendroglial density in the prefrontal cortex in schizophrenia and mood disorders: a study from the Stanley Neuropathology Consortium. Schizophrenia Research. 2004;67(2-3):269-75. doi: 10.1016/S0920-9964(03)00181-6. PubMed PMID: ISI:000220007500018.

54. Edgar N, Sibille E. A putative functional role for oligodendrocytes in mood regulation. Translational Psychiatry. 2012;2. doi: ARTN e109

10.1038/tp.2012.34. PubMed PMID: ISI:000312895700002.

55. Klugmann M, Symes CW, Klaussner BK, Leichtlein CB, Serikawa T, Young D, et al. Identification and distribution of aspartoacylase in the postnatal rat brain. Neuroreport. 2003;14(14):1837-40. doi: 10.1097/01.wnr.0000090955.15465.dd. PubMed PMID: ISI:000186291100016.

56. Moffett JR, Arun P, Ariyannur PS, Garbern JY, Jacobowitz DM, Namboodiri AMA. Extensive Aspartoacylase Expression in the Rat Central Nervous System. Glia. 2011;59(10):1414-34. doi: 10.1002/glia.21186. PubMed PMID: ISI:000294178300003.

57. Tallan HH. Studies on the Distribution of N-Acetyl-L-Aspartic Acid in Brain. Journal of Biological Chemistry. 1957;224(1):41-5. PubMed PMID: ISI:A1957WE89000004.

58. Urenjak J, Williams SR, Gadian DG, Noble M. Specific Expression of N-Acetylaspartate in Neurons, Oligodendrocyte-Type-2 Astrocyte Progenitors, and Immature Oligodendrocytes Invitro. J Neurochem. 1992;59(1):55-61. doi: DOI 10.1111/j.1471-4159.1992.tb08875.x. PubMed PMID: ISI:A1992HZ29000008.

59. Blume J, Douglas SD, Evans DL. Immune suppression and immune activation in depression. Brain Behavior and Immunity. 2011;25(2):221-9. doi: 10.1016/j.bbi.2010.10.008. PubMed PMID: ISI:000286699500003.

60. De Berardis D, Conti CMV, Serroni N, Moschetta FS, Olivieri L, Carano A, et al. The Effect of Newer Serotonin-Noradrenalin Antidepressants on Cytokine Production: A Review of the Current Literature. International Journal of Immunopathology and Pharmacology. 2010;23(2):417-22. doi: Doi 10.1177/039463201002300204. PubMed PMID: ISI:000280272600004.

61. Miller AH, Maletic V, Raison CL. Inflammation and Its Discontents: The Role of Cytokines in the Pathophysiology of Major Depression. Biological Psychiatry. 2009;65(9):732-41. doi: 10.1016/j.biopsych.2008.11.029. PubMed PMID: ISI:000265239400003.

62. di Penta A, Moreno B, Reix S, Fernandez-Diez B, Villanueva M, Errea O, et al. Oxidative Stress and Proinflammatory Cytokines Contribute to Demyelination and Axonal Damage in a Cerebellar Culture Model of Neuroinflammation. PLoS One. 2013;8(2). doi: ARTN e54722

10.1371/journal.pone.0054722. PubMed PMID: ISI:000315182800003.

63. Haroon E, Miller AH. Inflammation Effects on Brain Glutamate in Depression: Mechanistic Considerations and Treatment Implications. Curr Top Behav Neurosci. 2017;31:173-98. Epub 2016/11/11. doi: 10.1007/7854_2016_40. PubMed PMID: 27830574.

64. Ho PS, Yen CH, Chen CY, Huang SY, Liang CS. Changes in cytokine and chemokine expression distinguish dysthymic disorder from major depression and healthy controls. Psychiatry Res. 2017;248:20-7. Epub 2016/12/20. doi: 10.1016/j.psychres.2016.12.014. PubMed PMID: 27992767.

65. Leighton SP, Nerurkar L, Krishnadas R, Johnman C, Graham GJ, Cavanagh J. Chemokines in depression in health and in inflammatory illness: a systematic review and meta-analysis. Mol Psychiatry. 2017. Epub 2017/11/15. doi: 10.1038/mp.2017.205. PubMed PMID: 29133955.

66. Krishnadas R, Cavanagh J. Depression: an inflammatory illness? J Neurol Neurosurg Psychiatry. 2012;83(5):495-502. Epub 2012/03/17. doi: 10.1136/jnnp-2011-301779. PubMed PMID: 22423117.

67. Hiles SA, Baker AL, de Malmanche T, Attia J. A meta-analysis of differences in IL-6 and IL-10 between people with and without depression: Exploring the causes of heterogeneity. Brain Behavior and Immunity. 2012;26(7):1180-8. doi: 10.1016/j.bbi.2012.06.001. PubMed PMID: ISI:000308899600021.

68. Slavich GM, Irwin MR. From Stress to Inflammation and Major Depressive Disorder: A Social Signal Transduction Theory of Depression. Psychological Bulletin. 2014;140(3):774-815. doi: 10.1037/a0035302. PubMed PMID: ISI:000335224800007.

69. Kettenmann H, Ransom BR. Neuroglia. 2nd ed. New York: Oxford University Press; 2005. xix, 601 p. p.

70. Laping NJ, Teter B, Nichols NR, Rozovsky I, Finch CE. Glial Fibrillary Acidic Protein - Regulation by Hormones, Cytokines, and Growth-Factors (Vol 4, Pg 259, 1994). Brain Pathology. 1995;5(1):120-1. PubMed PMID: ISI:A1995QJ68400016.

71. Nair A, Bonneau RH. Stress-induced elevation of glucocorticoids increases microglia proliferation through NMDA receptor activation. Journal of neuroimmunology. 2006;171(1-2):72-85. doi: 10.1016/j.jneuroim.2005.09.012. PubMed PMID: ISI:000235114500008.

72. Reuss B, Unsicker K. Survival and differentiation of dopaminergic mesencephalic neurons are promoted by dopamine-mediated induction of FGF-2 in striatal astroglial cells. Molecular and Cellular Neuroscience. 2000;16(6):781-92. doi: 10.1006/mcne.2000.0906. PubMed PMID: ISI:000166190400007.

73. Chang L, Munsaka SM, Kraft-Terry S, Ernst T. Magnetic resonance spectroscopy to assess neuroinflammation and neuropathic pain. J Neuroimmune Pharmacol. 2013;8(3):576-93. Epub 2013/05/15. doi: 10.1007/s11481-013-9460-x. PubMed PMID: 23666436; PubMed Central PMCID: PMC3698315.
