# Supplementary material for: Effects of the antidepressant medication duloxetine on brain metabolites in persistent depressive disorder: A randomized, controlled trial
Source: PLoS One. 2019 Jul 19;14(7):e0219679. doi: 10.1371/journal.pone.0219679 (PMC6641507; doi:10.1371/journal.pone.0219679)
Supplement: S6 File — (PDF) [file pone.0219679.s017.pdf]

NEW YORK STATE PSYCHIATRIC INSTITUTE

INSTITUTIONAL REVIEW BOARD

MEMORANDUM

March 4, 2013

TO: Dr. David Hellerstein

FROM: Dr. Edward Nunes, Chairman, IRB

SUBJECT: **APPROVAL NOTICE: CONTINUATION APPROVAL**
Expedited per 45CFR46.110(b)(1)(f)(8)(c)

Your protocol **#6363R** entitled **DULOXETINE VS PLACEBO FOR CHRONIC DEPRESSION: A DOUBLE BLIND STUDY AND MRI SUB-STUDY** and Consent Forms (if applicable) have been approved by the New York State Psychiatric Institute - Columbia University Department of Psychiatry Institutional Review Board from **APRIL 4, 2013** to **APRIL 3, 2014.**

Consent requirements:

X Not applicable (RECRUITMENT COMPLETED. DATA BEING ANALYZED)

Signature by the person(s) obtaining consent is required to document the consent process.

Documentation of an independent assessment of the participant's capacity to consent is also required.

Approved for recruitment of subjects who lack capacity to consent: No Yes

Field Monitoring Requirements: Routine Special:

√ **Only copies of consent documents that are currently approved and stamped by the IRB may be used to obtain consent for participation in this study.**

√ **A progress report and application for continuing review is required 2 months prior to the expiration date of IRB approval.**

√ **Changes to this research may be not be initiated without the review and approval of the IRB except when necessary to eliminate immediate hazards to participants.**

√ **All serious and/or unanticipated problems involving risks to subjects or others must be reported immediately to the IRB. Please refer to the PI-IRB website at http://irb.nyspi.org for Adverse Event Reporting Procedures and additional reporting requirements.**

CC: RFMH (Eli Lilly)

ls

Protocol Title:
Duloxetine vs. Placebo for Chronic Depression: A Double-Blind Study and MRI Sub-Study (Previously #4967)

Version Date:
03/04/2013

Protocol Number:
6363R

First Approval:
04/18/2011

Expiration Date:
04/03/2013

Principal Investigator:
David Hellerstein, MD
Email: hellers@pi.cpmc.columbia.edu
Telephone: 212-305-1415

Co-Investigator(s):
Patrick McGrath, MD
Jonathan Stewart, MD
Deborah Deliyannides, MD

Status

Status

Current Status of Study:
Study has only involved data analysis or closed record review of data in the past year, or study has only involved the study of existing data.

Are you proposing a study modification with this application?
No

Funding

Funding

Have there been any changes in funding status since the prior approval?
No
Have the principal investigator and other investigators made all required disclosures of financial interest in the study sponsor/product?
Yes

Principal Investigator:
Hellerstein, David, MD
Title of currently funded grant:
Duloxetine vs. Placebo for Chronic Depression: A Double-Blind Study and MRI Sub-Study
Source of funding:
Eli Lilly Co.
Grants office:
RFMH

Summary

Summary

Have there been any study findings, recent literature, or untoward events occuring here or at other sites in the past year which might affect the analysis of the safety, risks or benefits of study participation?
No
Have there been any serious adverse events (serious and/or unanticipated problems involving risks to subjects or others at this site which occured in the past year)?
No
Have all study staff with a significant role in the design or implementation of the human subject components of this study received required training in human research subject protections?
Yes
Is the study covered by a certificate of confidentiality?
No

Accrual

Approved Sample and Progress

Approved sample size
80
Total number of subjects studied since first approval
65
Have there been any significant deviations from the anticipated study completion estimates?
No
Comments / additional information

Uploads

You have indicated that you are not proposing a modification. Please upload the current, unchanged Protocol Summary Form.
duloxetine_PSF_rev-3-14-2012-PDF.pdf
Please upload any additional documents if applicable

NEW YORK STATE PSYCHIATRIC INSTITUTE
INSTITUTIONAL REVIEW BOARD

PROTOCOL SUMMARY FORM (v.4/10)

IRB# 4967

Version Date: 3/14/12

Title of Proposed Study: Duloxetine for chronic depression: a double blind study

Name and Title of Principal Investigator: David J Hellerstein, MD

Research Department: Depression Evaluation Service

Telephone: 212-543-5743

Email: hellers@pi.cpmc.columbia.edu

List Co-Investigators (or Sponsor if principal investigator is a research fellow or trainee):

A. Jonathan W. Stewart MD B. Patrick McGrath, MD C. Deborah Deliyannides, MD

D. E. F.

Sites as which study procedures will be conducted (check all that apply):

__X_P.I. ___NYPH/CUMC ___CU downtown

___Other OMH facility (specify):

___Community (specify):

__X_Other Institutions/facilities (specify):______3 Columbus Circle, NY NY

Funding Information:

__X__Funding active _____Funding pending ____No funding

Indicate grants office:

___CU_X__RFMH

Source of funding:

____Federal ____Foundation __x__ Industry/Pharmaceutical ____Internal ____Other

Source of funding (specify name or agency/institute and grant number):

If industry supported: Eli Lilly, Co. Duloxetine for chronic depression: a double-blind study.

Protocol #F1J-US-X001

___Sponsor-initiated __x_Investigator Initiated

I. Abstract: Describe objectives and methods of study. Write in layman's language, without jargon, technical terms or undefined abbreviations. Do not exceed one page.

This is a 22-week study of the tolerability, dosing, and efficacy of duloxetine in chronically depressed outpatients. The first 10 weeks (Acute Phase) are double blind, placebo-controlled, and the second 12 weeks (Continuation Phase) is open-label and all subjects will receive active medication. Tests of cytokine functioning will be performed and analyzed for treatment and placebo effects. In addition, a subset of patients will be enrolled into an MRI substudy in which MRI analyses (including anatomical MRI, fMRI, MR Spectroscopy, and Diffusion Tensor Imaging) of brain anatomy and functioning will be performed at baseline and week 10. Duloxetine responders will have a third MRI performed at week 22. For the purposes of distinguishing details and criteria relating to the overall study from the separate MRI details and criteria, hereafter the overall study will be called The Main Study and the MRI subset will be called The MRI Study.

II. List names and titles of persons designated to obtain consent.

David Hellerstein MD, Patrick McGrath, MD, Jonathan Stewart MD, Deborah Deliyannides, MD

III. Research Plan:

1. <u>Hypotheses to be tested:</u> If there are no specific hypotheses, describe study goals.

<u>Efficacy Hypotheses:</u>

1. It is hypothesized that duloxetine will be superior to placebo in improving depression, as measured by the HDRS-24 item total score at week 10.
2. It is hypothesized that duloxetine will be superior to placebo in the percentage of subjects classified as (a) Responders and (b) Remitters at week 10
3. It is hypothesized that duloxetine will be superior to placebo in improving secondary measures of depression (CDRS, SCL-90-R), and measures of psychosocial, temperamental, pain, and cognitive functioning (GAFS, CGI-Severity, CGI-Improvement, BDI, TCI, BPI, SAS, MOTCS) at week 10
4. It is hypothesized that at week 22: (a) patients who continue duloxetine treatment to week 22 will show continued positive response, and (b) patients who were on placebo to week 10 and begin active medication will show significant improvement.

<u>Biological Hypotheses:</u>

1. The levels of IL-1□, IL-6, and TNF□ cytokines, and a.m. cortisol will be abnormally high at baseline in chronically depressed subjects,
2. IL-1□, IL-6, and TNF□ cytokine elevations will persist at 10 weeks in placebo-treated subjects,
3. 10 weeks of duloxetine treatment will lead to significant decrease in IL-1□, IL-6, and TNF□ cytokine levels compared to baseline levels but which will remain higher than normal reference samples,
4. After 22 weeks of duloxetine treatment, IL-1□, IL-6, and TNF□ cytokine levels and a.m. cortisol levels in medication-treated subjects will drop significantly compared to baseline levels,

5. A positive association will exist between depressive symptomatology (severity, remission status) and cytokine and cortisol elevations, both at baseline, at week 10, and at week 22,
6. The level of cytokine IL-10 will be normal at baseline in chronically depressed subjects, and will be equivalent for placebo and duloxetine treated subjects at week 10, and at week 22,
7. Subjects with higher baseline levels of cytokines and cortisol will show poorer response to treatment with duloxetine.

MRI Hypotheses:
1. Anatomical MRI
a. Chronic depression vs. normal differences: we expect to find abnormal brain region volumes in several regions compared to norms. Specifically: Frontal Cortex: decreased dorsal prefrontal volume, and reduced grey matter in subgenual prefrontal cortex; reduced orbitofrontal cortex gray matter volume; decreased hippocampal volume; decreased amygdala volume; decreased caudate volume; decreased putamen volume
b. treatment vs. placebo differences: following active treatment, we hypothesize that volumes will change in the direction of normal in the hippocampus, prefrontal cortex, subgenual cortex, and amygdala
c. responder vs. non-responder differences: greater changes in the areas found in hypothesis 2 are expected in treatment responders compared to non-responders

2. Diffusion Tensor Imaging (DTI):
a. We hypothesize that there will be reduced fractional isotropy in prefrontal cortices in the depressed group compared to normals, and we hypothesize that this will normalize with treatment.

3. fMRI
a. Simon Task:
i. We expect reduced frontal cortex activation in depressed subjects, and
ii. normalization of frontal cortex activation following treatment.
b. Affective Circumplex Task:
i. Reduced amygdala and hippocampal activation in depressed subjects,
ii. normalization of activation in these areas following treatment.

4. MR Spectroscopy
We expect to find decreased levels of NAA (N-acetyl-aspartate) in frontal cortex (dorsal prefrontal, subgenual prefrontal cortex, and orbitofrontal cortex gray matter), as well as in hippocampus, amygdala, caudate, and putamen

2. Recruitment Methods

A. Recruitment Methods and Description of Approach to Research Subjects: Attach to this form any letters to be sent, texts of advertisements, etc., if available now. If not available now, they must be submitted to the IRB prior to the initiation of recruitment procedures.

Subjects will be recruited using advertisements in local newspapers, advertisements and notices on internet sites such as Centerwatch.com, Clinicaltrials.gov, DepressionNY.com, ColumbiaPsychiatry.org, and by soliciting clinicians in the area.

B. If subjects from other studies are to be asked to participate, list studies with their IRB #, principal investigator and title.

Gerard Bruder, Ph.D.: Behavior ERP and EEG Asymmetry in Affective Disorders (IRB# 4416R)

Dev Devanand, MD.: MR indicators of antidepressant treatment response and neurogenesis (IRB# 5450)
David Hellerstein, MD: Behavioral Activation (BA) For Medication-Responsive Chronically Depressed Patients With Impaired Social Functioning (IRB#5908)

C. Subjects: Specify sample sizes, sex, ethnicity, age range, diagnostic group and other relevant characteristics.

The composition of the proposed study population must be described (by number or percentage) in terms of (a) gender and (b) racial/ethnic group. If one gender and/or minorities are excluded or inadequately represented, a rationale should be provided.

N (for recruitment)= 80
N (completers)= 65
Age range: ___20____ to ____75__

Sample Description:

Main Study

80 adults, aged 20-75, with, Dysthymic Disorder, or Depressive disorder NOS as defined by DSM-IV with a duration of 24 months or longer. No racial, ethnic, or cultural groups are excluded. It is expected that the sample will approximately 50% male and 70% Caucasian, based on previous studies at our research unit. Other ethnic groups (e.g. Asian, Hispanic, African-American) have each comprised between 5-10% of our previous studies.

MRI Study

40 to 50 subjects enrolled in the Main Study will be entered into the MRI Study. We expect no difference in the percentage of any ethnic or gender group in the MRI Study as compared to the larger Main Study. The MRI sample will be younger, as the age range will be 20-60.

3. STUDY INCLUSION AND EXCLUSION CRITERIA (list in outline form)

CRITERION **METHOD OF ASCERTAINMENT**

**Inclusion Criterion**	**Method of Ascertainment**
Main Study	
1. Male and female outpatients 20 to 75 years of age, inclusive	Self-report from interview with patient
2. Patients with a principal DSM-IV diagnosis of Dysthymic Disorder, or Depressive Disorder NOS	SCID Interview at screening visit

3. a minimum of 2 years duration of the current episode of depressive disorder.	Patient self-report at screening in SCID interview
4. Patients will have a total of 12 or higher on the Hamilton Depression Scale (24 items) at baseline	HDRS-24 total score at baseline visit
5. Patients agree not to take any other psychotropic medications through the course of the study, including herbal preparations with putative psychotropic effects	Patient self report at screening visit
6. Women of childbearing potential must agree to use acceptable means of birth control throughout the study.	Patient self-report at screening.
MRI Study Inclusion criteria:	
1. Male and female outpatients between the ages of 20 and 60 years, inclusive.	Patient self report at screening visit
2. Individuals must provide informed consent for MRI scanning	Patient self report at screening visit

Exclusion:

Exclusion Criteria	Method of Ascertainment
Main Study	
1. Patients who have had a full remission of depressive symptoms, as defined by DSM-IV, at any time within the past 24 months	SCID interview
2. Patients with any of the following DSM-IV diagnoses:<br>a) Major Depressive Disorder<br>b) Delirium, Dementia, Amnestic, or other Cognitive Disorders.<br>c) Bipolar Disorder or Cyclothymia, Schizophrenia, Delusional (Paranoid) Disorders and Psychotic Disorders not elsewhere classified, Severe Borderline Personality Disorder, Anorexia Nervosa, or Bulimia.	Clinician judgment based on<br>a) clinician judgment after medical and psychiatric history interview<br>b) SCID Interview
3. Patients with additional comorbid psychiatric conditions of moderate or greater intensity (such as PTSD, OCD. Agoraphobia, Panic Disorder) which would cause risk or suffering to the patient if left untreated	Clinician judgment based on<br>a) clinician judgment after medical and psychiatric history interview<br>b) SCID Interview
4. Patients who, within the past 6 months, met DSM-IV criteria for abuse of or dependence on any drug, including alcohol.	SCID interview, urine drug test at screening
5. Patients who are pregnant or nursing women.	Patient self-report, pregnancy test at screening

6. Patients who would pose a serious risk for suicide during the course of the study, as evidenced by one of the following: (a) report of having a specific plan for killing themselves, (b) a score of 3 or higher on the Hamilton Depression Rating Scale item #3 as rated by the treating clinician at Week 0, or (c) a suicide attempt within the last 6 months which required medical attention, such as an emergency room visit or which is considered by the treating physician to have been possibly life threatening	SCID interview at screening, HDRS ratings from screen and/or baseline, patient self-report, clinician judgment
7. use of any psychotropic medication within 1 week of starting study medication	Patient self-report
8. Use of an Monoamine oxidase inhibitor (MAOI) within the 14 days prior to the initial dose of study medication	Patient self-report
9. Use of fluoxetine within 28 days (4 weeks) of the initial dose of study medication	Patient self-report
10. Patients who are "treatment-resistant" as defined by failure to respond to adequate trials (minimum of 6 consecutive weeks) of two different classes of antidepressant medication (see **Table 1** for definitions of Adequate Trial) within the last two years	Medical history interview by MD, SCID interview at screening, and/or patient self-report
11. Patients with mild dysthymic disorder, as defined by HDRS-24 score of 11 or less	HDRS-24 score at baseline
12. Patients with unstable medical conditions such as untreated or uncontrolled hyperthyroidism, hypothyroidism, hypertension, diabetes, significant cardiac conduction abnormality on EKG**,** or HIV (by report of patient).	Medical history interview by MD, blood and urine test results at screening, patient self-report, physical exam
13. Patients who are hypersensitive or allergic to duloxetine	Medical history interview by MD, and/or patient self-report
14. Patients with the following medical conditions in which duloxetine in contraindicated or in which metabolism and clearance is markedly affected:<br>a) Uncontrolled narrow angle glaucoma<br>b) hepatic insufficiency<br>c) end stage renal disease<br>d) patients on arrhythmia medications<br>e) history of a seizure disorder<br>f) any medical condition that significantly slows gastric emptying	Medical history interview by MD, blood and urine test results at screening, patient self-report
15. Patients who have not responded to a previous adequate trial of duloxetine (e.g. 60 mg for 6 weeks without significant improvement of depressive symptoms)	Patient self report at screening
MRI Study exclusion criteria	

1. A history of blunt head trauma	Medical history interview by MD, patient self-report, physical exam
2. History of surgery on the brain or skull	Medical history interview by MD, patient self-report, physical exam
3. Current diagnosis of significant neurological disease, including lifetime history of epilepsy or other seizure disorder	Medical history interview by MD, patient self-report, physical exam
4. Patients with pacemakers or other metal objects in their bodies (e.g. shrapnel, bullets, implants, surgical clips, braces, IUDs, nicotine or other medicinal patches that cannot or will not be removed during scanning)	Patient self-report, medical history interview by MD, interview with MRI personnel prior to scan, NYSPI metal Screening Questionnaire
5. Patients for whom MRIs are contraindicated for any other reason, including significant claustrophobia	Medical history interview by MD, investigator judgment,
6. Patients who have taken psychiatric medication within 4 weeks (28 days) of the baseline MRI	Patient self report at screening
7. Pregnant or nursing women	Urine pregnancy tests prior to each MRI scan

Table 1. Acceptable SSRI Antidepressants and Adequate Dose

Antidepressant	Adequate dose (mg/day)
SRIs	
Citalopram	40
Fluvoxamine	150
Paroxetine	40
Sertraline	150
Fluoxetine	40
Escitalopram	20
SNRIs	
Venlafaxine XR	225
Duloxetine	60

4. <u>STUDY PROCEDURES</u>: Please provide a flow-chart (diagram) of study procedures.

A. Provide details of all procedures including credentials (R.N., M.D.) of person(s) conducting each procedure including interviews.

If medication is to be used, specify dose and dose schedule. For more complex designs, flow diagrams are helpful.

MAIN STUDY

Screening visit/Visit 1:

Prospective patients will be assessed on the following

- SCID-P I and SCID-II Borderline module for diagnostic inclusion and exclusion criteria by trained SCID interviewers
- psychiatric and medical history will be assessed by a psychiatrist,
- physical exam
- blood and urine will be collected and analyzed, and
- patients will complete a set of questionnaires assessing symptoms
- EKG will be done for individuals age 60 and above.

For patients undergoing a washout period prior to starting study medication, the study doctor assigned to the patient will schedule weekly visits with the patient during the taper period. If weekly visits are declined, the study doctor will make weekly phone calls to the patient to monitor the withdrawal from the previous medication. If patients followed by telephone while being discontinued from medication express significant distress they will be asked to come to the Clinic to be evaluated. The length of the washout period will depend on the medication being discontinued. See the “Prior Medications” section for length of washout period required.

Baseline/Visit 2/ Week 0:
Eligible subjects will return 1 week later to meet with a psychiatrist who will administer rating scales. If HDRS score criteria are met, patients will complete additional questionnaires assessing symptoms and social functioning. Patients will then begin 10-weeks of double-blind study drug.

Visits 3 (week 1) through 5 (week 4):
Patients will be assessed by a psychiatrist regularly throughout the trial and will return for subsequent Acute Phase evaluations at weeks 1, 2, 4. At these evaluations the patient’s symptoms, level of functioning, adverse experiences, concomitant medications, and vital signs will be assessed and recorded. At that time, (a), the treating psychiatrist administers rating scales, (b) patients will complete self-report questionnaires regarding their symptoms and functioning, (c) study personnel will assess and record vital signs, concomitant medications, and adverse experiences.

Week 6 (Visit 6)

At week 6, patients will be evaluated using the same measures as weeks 0 through 4. Based on this week’s CGI-Improvement score and their history of CGI-Improvement scores at previous visits, their continuing participation will be determined based on the following decision-tree (Decision Tree, **Table 2**). Patients continuing in the study with CGI-I scores of 4 or 5 will be reconsented at this visit and this will be documented in their record.

Table 2: Decision-tree

CGI-I at week 6	CGI-I history	Required actions and evaluations
1, 2, or 3	Any	Continue in Acute phase, perform week 6 evaluations
4 or 5	Any CGI-I < 4	Reconsent patient, perform week 6 evaluations, and continue in Acute Phase
4 or 5	All CGI-I $\geq$ 4	Perform Final Acute Phase evaluations (including collecting blood sample), unblind patient and enter into Continuation Phase
6 or 7	See safety section for how to proceed.	

Visit 7/ Week 8

Again at week 8, if the patient's CGI-Improvement score is 4 or higher, the patient will be reconsented and this consent will be documented in the patient's chart. Week 10 (Final Acute phase) evaluations will be performed for patients who do not consent to continue. All others will be evaluated according to week 8 procedures.

Visit 8 / Week 10: *Final Acute Phase Visit*

In addition to the week 10 assessments, blood will be drawn for cytokine (IL-6, plasma IL-1β, TNFα, and IL-10) and cortisol analysis, and questionnaires assessing social functioning, temperament, cognitive functioning will be completed by the patient. The subject's study drug assignment will be unblinded by a second psychiatrist (who will assume treatment of the patient in the Continuation Phase) and the patient will continue into the Continuation Phase for 12 weeks of open-label treatment. If the patient was on placebo they will be started on duloxetine as per the acute phase drug dosing schedule. For subjects who are responders to placebo, study physician will have a discussion of the placebo response and the pros and cons of taking antidepressant medication before offering them duloxetine in the continuation phase. If the patient had been taking duloxetine and was a non-responder after the first 10 weeks, they will be switched to another FDA approved antidepressant medication after an appropriate withdrawal period. (See **Table 3**). For patients who are switching from duloxetine to another medication, duloxetine dosage will be tapered at a rate of 30 mg approximately every 4 days, or as clinically tolerated.

Table 3. Continuation medication options.

Treatment Condition	Treatment Response	Continuation Phase medication
Placebo	Responder	Any FDA approved antidepressant
	Non-Responder	Duloxetine
Duloxetine	Responder	Duloxetine
	Non-responder	Any FDA approved antidepressant

Visits 9 through 13 (weeks 11 through 18):

At weeks 11, 12, 14, 16, and 18, visits will be conducted similar to visits 3-7. A psychiatrist will evaluate the patient's symptoms and level of functioning and these will be assessed and recorded in the following ways: (a) the treating psychiatrist administers rating scales, (b) patients will complete self-report questionnaires regarding their symptoms and functioning, (c) study personnel will assess and record vital signs, concomitant medications, and adverse experiences.

Visit 14 (week 22, end of Continuation Phase):
In addition to the assessments normally performed at visits 9 through 13, blood will be collected for cytokine and cortisol analysis, and self-report questionnaires assessing social functioning, temperament, and cognitive functioning will be completed. Tapering off duloxetine will begin at this visit for patients discontinuing use of the medication. The dose will be tapered at a rate of 30 mg approximately every 4 days, or as clinically tolerated. For patients continuing medications, appropriate referrals will be provided.

MRI STUDY

Screening

Patients will be consented separately for the MRI study at screening. Those who consent will be evaluated for MRI inclusion and exclusion criteria by the study physician at screening as well as at each MRI visit by the MRI personnel. Patients who meet these criteria will be scheduled for an appointment at the MRI clinic at NY State Psychiatric Institute.

General MRI procedure:

The subject will be instructed to lie as still as possible within the magnet for approximately 75 minutes. All precautions and protections will be given to the participant to ensure that they are as safe and comfortable as possible. For the participant's comfort within the scanner, they will lie on a padded table with a pillow to rest their heads on. A blanket will also be provided to keep subjects warm during the procedure. If the participant appears nervous or anxious, a trained member of the clinical staff will remain with them inside the scanning suite for the duration of the scan. The participant will be given a button box to terminate the scan at any time. If they push the button, they will be removed from the scanner immediately. If they choose not to finish the scan, they will still be eligible to participate in the main study. All of the MRI procedures will be conducted on the 3-Tesla MRI scanner at the New York State Psychiatric Institute. Conducting these procedures will be an accredited Magnetic Resonance Technologist (B.M.R.) and a member of the clinical staff (Bachelor's Level or Higher) trained in the acquisition of MR images by Dr.Peterson, as well as in procedures for testing human subjects. Although our MRI Scans are for research purposes, a radiologist will perform a clinical reading on every MRI within 1 month of scanning; if anything clinically significant is found, Dr. Hellerstein or an appropriately trained clinician will be notified immediately, and this clinician will provide an appropriate clinical referral to the participant.

MRI Protocol

Pulse Sequence	Scan Time (Approximate)
3-Plane Localizer	.5 minutes
Anatomical Sequence	10 minutes
DTI Sequences	10 minutes
Functional Images<br>• Simon Spatial Compatibility Task<br>• Affective Circumplex Task	30 minutes
MR Spectroscopy	20 minutes

Patients will spend approximately one to one and one half hours in the MRI machine, and four types of MRI data will be obtained:

1. MRI of brain structure (specifically the prefrontal cortex, hippocampus, amygdala, and anterior cingulate cortex);

2. functional MRI will be performed during which subjects will perform two structured tasks, the Simon Spatial Incompatibility Task, and the Affective Circumplex Task using both semantic and facial cues.

i. Affective Circumplex Task

a) Semantic Cues

Subjects will rate the similarity/dissimilarity of pair-wise combinations of 16 affective terms. Using multidimensional scaling, a geometric representation will be obtained for each term. Similar scaling procedures have been used behaviorally by many researchers (Kring, A.M., et al., 2003; Russell, J.A., 1980) yielding a 2 dimensional structure with each term defined by the degree of arousal and valence. We will parametrically correlate the imaging characteristics generated as the subjects evaluate the terms with the geometric values obtained for each term from the scaling procedure. Additionally, subjects will be asked to rate each term individually. Subjects will rate each term according to its valence and arousal. The terms to be used are: *excited*, *lively*, *cheerful*, *pleased*, *calm*, *relaxed*, *idle*, *still*, *dulled*, *bored*, *unhappy*, *disappointed*, *nervous*, *fearful*, *alert*, and *aroused*

b) Facial Expressions Cues

Subjects will rate the similarity/dissimilarity of pair-wise combinations of emotionally expressive faces. Subjects will also assess each facial expression individually, rating the emotion expressed by each facial expression according to its valence and arousal. In a similar manner to the semantic approach, correlations will be made with image intensities. The facial expressions to be used are from Ekman (1976) and Russell & Bullock (1986).

ii. The Simon Spatial Incompatibility Task

In this task leftward- or rightward-pointing arrows are presented on either side of a screen. Subjects are instructed to press a button with either their first or second finger of their right hand to indicate whether the arrow is on the right or left side of the midline. Most of the arrows presented (on average 95%) are congruent stimuli (e.g. a right-pointing arrow is shown on the right side of the screen), but incongruent stimuli (e.g. a right-pointing arrow is shown on the left side of the screen) are randomly interspersed among them. Responses to the incongruent stimuli are markedly slow relative to responses to congruent stimuli in normal subjects. The duration and number of stimuli are identical to those used in the Stroop, a well-documented interference task. Publications from Peterson et al. indicate that the brain regions that are engaged in resolving the interference effects of the incongruent stimuli are similar to those engaged by the Stroop Word-Color Interference Task. The Simon therefore appears to be a spatial analogue of the Stroop Task. We prefer to use the Simon as it will avoid the possible confounds posed by reading or language difficulties. Furthermore, the Simon also allows for online monitoring of task performance during the scan.

3. Diffusion Tensor Imaging (DTI) to obtain information regarding the health of nerve tracts.

4. MR Spectroscopy to evaluate the chemical composition and integrity of neurons in the brain

MRI visit 1
This appointment will take place within 1 week of the screening visit, and will occur before beginning study medication. The NYSPI – Dept. of Psychiatry MRI Metal Screening Questionnaire will be administered. This is a 38-item questionnaire that asks specifically about metallic implants and past experiences with metal to further ascertain any possible risks the person may incur by entering the scanner. Again, if any metallic implants or medicinal patches they do not wish to remove that are unsuitable for the scanner are detected, the subject will not be included in the MRI portion of our study.

MRI visit 2
Subjects will return approximately 10 weeks later (after completion of the Acute Phase), will complete the Metal Screening questionnaire, and eligible subjects will then undergo a second MRI scan using the same MRI protocol as at week 0.

MRI visit 3
Eligible subjects (duloxetine responders) will return for a third scan approximately 12 weeks subsequent to their second scan (after completion of the continuation phase), will complete the Metal Screening Questionnaire, and eligible subjects will undergo the MRI protocol.
Women who are capable of becoming pregnant will have urine pregnancy tests performed within 7 days prior to having an MRI scan. A positive pregnancy test will exclude the subject from participating; no scan will be performed on a pregnant woman.

Study Medication

Acute Phase

Double-blind treatment with duloxetine or placebo will occur in the Acute Phase, weeks 0 to 10. Initial doses will be 30 mg duloxetine or placebo for 1 week. Patients who have difficulty tolerating this starting dose may take 30 mg every other day until this dose is well tolerated. Once the dose is tolerated attempts to increase dose to 60 mg/day will be made.
Subesequent to starting 60 mg per day, dose increases of 30 mg per day will be made every 4 weeks if current dose is well tolerated and CGI-I scores indicate minimal or no improvement (CGI-I >= 3). Maximum dose will be 120 mg/day. Placebo pills will be identical and follow the same dosing schedule.

Continuation Phase

At week 10, patients will be switched to a new doctor who will break the blind and begin 12 weeks of open-label treatment with either duloxetine or another FDA approved antidepressant. (see **Table 3**)

Prior Medications:

No other psychotropic medications will be allowed within one week of starting study drug. The minimum washout period for excluded medications is 7 days. The washout period for fluoxetine is 28 days, and the washout period for MAOIs is 14 days.

Concomitant Medications

Patients will not be allowed to take any other psychotropic medication during the course of the study, including benzodiazepines, barbituates, narcotics, or herbal preparations with putative psychotropic or sleep inducing effects (e.g. St. Johns Wort, kava-kava, valerian, SAMe). No hypnotic medications or over-the-counter sleep aids will be allowed after visit 1, with the exception of Zolpidem if needed for insomnia after visit 2. Zolpidem may be titrated to a maximal dose of 10 mg/HS. Use of Zolpidem may not exceed 6 total days during each phase.

(see **Table 4**, Schedule of Events)

B. What is maximum duration of delay due to research procedures before patient begins a treatment of any kind?

Most patients will have one week between evaluation/screen and the onset of double blind medication. 4 weeks is the maximum time between evaluation and starting active treatment (for those patients entering the study while taking fluoxetine and are required to have a 4 week washout period).

C. What is maximum duration of delay before active treatment (medication or psychotherapy) that is part of routine care or of known efficacy is offered? (Include time period described in B. above.)

The drug under study is currently approved by the FDA for treatment of Major Depressive Disorder. While it has been shown effective for this, it has not yet been demonstrated to be effective for chronic forms of depression. (No drug has been approved for chronic depression, although dual mechanism medications, such as duloxetine or venlafaxine, are thought to have greater efficacy than single mechanism medications for various forms of depression, including chronic depression). Therefore, whether this medication has known efficacy for chronic depression is difficult to state with precise certainty at this time. If the drug can be considered efficacious, then 50% of the patients will receive a known efficacious drug within 1 week in general (and 4 weeks maximum) of starting the study and the other 50% will receive a drug with known efficacy after a delay of 11 weeks (1 week screening period and 10 weeks double blind treatment) with a maximum delay of 14 weeks for patient who are required to have a four week washout of fluoxetine prior to beginning double blind treatment. If it cannot be considered efficacious, then none of the patients will receive a known efficacious drug during the course of the study.

D. Describe treatment to be provided (if any, including duration) at the end of the study.

At the end of the study, we are offering three months of free continuation of treatment. All patients who reach 6 weeks, will be eligible for the continuation phase which consists of 3 months of open label treatment with an FDA approved antidepressant by the end of the double-blind portion of the study. Subjects who are withdrawn from the study by investigators before 6 weeks as a result of worsening of clinical status (as noted under "Procedures to Minimize Risk") will also be eligible for the continuation phase of open label treatment at no cost. Non-response is determined beginning at 6 weeks and those subjects are also eligible for continuation treatment. After the study, we will provide three months of free treatment including free antidepressant medication. After that time, we will make a referral to other doctors or clinics where ongoing treatment can be obtained. Since duloxetine has been approved by the FDA, it will be available to patients after the study is over.

E. If an experimental treatment will be used, describe other accepted/standard methods of treatment available, if any.

A variety of antidepressant medications are available for the treatment of depression, including SSRIs, SNRIs, tricyclics, and MAOIs. Psychotherapy, particularly cognitive-behavioral and interpersonal therapies, has been found to be effective for treatment of depression. In severe cases, ECT has been found effective. As mentioned above, the efficacy of all these treatments for chronic forms of depression is less well-established

5. <u>BLOOD SAMPLES (</u>State quantities to be drawn over what period and for what purpose).

Blood will be drawn three times during the study: at Screening, at visit 8, and at week 22 (or at the early discontinuation visit if the patient chooses to discontinue earlier than this). At screening, the amount of blood drawn is approximately 1 tablespoon or 15 ml. The reason for the blood draw is to evaluate patient safety and eligibility for the study using

lab tests such as CBC and differential, platelet count, Blood chemistry screen, Thyroid function (T3RU, T4RIA, TSH), and to test for cortisol and cytokine levels. At week 10 and 22, approximately 10 milliliters or 2 teaspoons of blood will be drawn for the purpose of measuring cytokine and cortisol levels.

6. **INSTRUMENTS: List measures to be used, including tests and interviews and time required for the completion of each. Attach copies unless standard instruments are used.**

Main Study

Depression Measures	
Hamilton Depression Rating Scale (HDRS-24 items)	20 minutes
Beck Depression Inventory (BDI)	5 minutes
Global Functioning Measures	
Clinical Global Impressions (CGI)	5 minutes
Global Assessment of Functioning Scale (GAFS)	5 minutes
Patient-CGI (CGI-P)	2 minutes
Social Functioning Measure	
Social Adjustment Scale (SAS)	10 minutes
Endicott Work Productivity Scale (EWPS)	5 minutes
Cognitive Behavioral Avoidance Scale (CBAS)	5 minutes
General Distress Measure	
Symptom Checklist (SCL-90-R)	10 minutes
Temperament Measure	
Temperament and Character Inventory (TCI)	25 minutes
Side Effects and Cognitive Functioning Measures	
Medical Outcomes Trust Cognitive Scale (MOTCS)	2 minutes
Aldenkamp-Baker Scale (ABS)	2 minutes
Arizona Sexual Experiences Scale (ASEX)	2 minutes
Pain Measure	
Brief Pain Inventory	2 minutes

MRI Study

MRI Metal Screening Questionnaire	5 minutes

The Metal Screening Questionnaire, a 38-item questionnaire that was developed by Dr. Peterson (as the Director of the MRI Unit), will be administered to every subject before they enter the MRI scanner. This questionnaire asks specifically about metallic implants and past experiences with metal to further ascertain any possible risks the person may

incur by entering the scanner. Again, if any metallic implants are detected that are unsuitable for the scanner, the subject will not be included in the MRI portion of our study. All precautions will be taken for the safety of the participants.

IV. Additional Information:

1. **Will subjects receive any compensation: YES__x_ NO___**

   If yes, how much?___Maximum of $505 (see below)
   (See Consent Form Guidelines re: payment procedures)

2. **Will procedures involve imaging studies and radio labelled compounds?**

   YES___ NO_x__

   If yes, what radiotracers?_imaging is done but no radiotracers are used_______

   Approval is required from the CU Joint Radiation Safety Committee.

3. **If investigational drugs are involved, complete the following:**

   1. **Generic or chemical name**
   2. **Other name**
   3. **Manufacturer**
   4. **IND #**
   5. **IND holder/sponsor**

4. **If an investigational device is involved, complete the following:**

   1. **Name**
   2. **Manufacturer**
   3. **IDE #**
   4. **IDE holder/sponsor**

5. **Describe risks to subjects and procedures for minimizing risks.**

Possible Risks:

Main Study:

The most common side effects seen in patients taking duloxetine are nausea, dry mouth, constipation, decreased appetite, fatigue, sleepiness, and increased sweating. Other side effects reported include diarrhea, vomiting, upset stomach, weight loss, dizziness, tremor, hot flushes, blurred vision, insomnia, nervousness, irritability, decreased sexual desire, abnormal orgasm, delayed or abnormal ejaculation, and small increases in blood pressure, and/or rash. A few patients have had nightmares while taking duloxetine or after stopping

duloxetine. Suicidal thoughts have also been noted as an 'infrequent' (less than 1%) occurrence.

The above side effects are expected to occur in some patients when they are taking medications of this type, but few patients have had to discontinue duloxetine because of side effects. Although studies have shown little effect of duloxetine on performance, it may have the potential to impair judgment, thinking or motor skills. Therefore, we will advise patients to use caution when performing potentially hazardous tasks such as operating an automobile or machinery until they can be reasonably certain that duloxetine does not interfere with their ability to do these tasks safely. It is possible, but unlikely, that a subject could be hypersensitive to duloxetine or that there could be some serious side effects that are still unknown to us.

<u>MRI Study:</u>

Both the FDA and the NSYPI IRB have deemed MRI Scanning on the GE 3Tesla MRI Scanner at the New York State Psychiatric Institute to be classified as a non-significant risk.

<u>Risks of Study Procedures</u>

(1) There may be unknown risks to an embryo, fetus, or unborn child of women taking this medication.
(2) Needle punctures for blood draws may cause bleeding, bruising, discomfort, dizziness, infections and/or pain at the needle site.
(3) Patients may experience a return or worsening of symptoms. It is also possible that duloxetine will have no benefit for subjects. (Symptoms which may worsen include: suicidal feelings or thoughts depressed mood, sadness, anhedonia, lack of motivation, difficulty concentrating or making decisions, insomnia (or hypersomnia), feelings of guilt and/or worthlessness, and or loss (or increase) of appetite).
(4) If patients stop taking any previous medications they may experience a return or worsening of symptoms (see above). This may also happen if they abruptly stop any of the study drugs anytime during the study.
(5) In addition to the known possible side effects, duloxetine or the study procedures may have other unknown risks.

<u>Procedures to Minimize Risks</u>

<u>Main Study</u>

- To closely monitor adverse events and symptom changes, patients are seen weekly at the start of each study phase when medications are initiated, and biweekly after that. To reduce the impact of side effects, flexible starting doses will be utilized to maximize tolerability in the early weeks of treatment.
- Patients are also instructed that they can call at any time to report adverse events.
- They are informed that they may withdraw consent at any time and that the investigator may withdraw them from the study if s/he feels that their safety is at risk.

- Patients are informed of all risks in the consent form.
- Pregnant and nursing women are excluded from the study, and use of an acceptable form of birth control is required for participation, to reduce risks to fetuses, unborn children, and nursing babies.
- Medication will be provided in childproof packaging designed to be difficult to open, in order to protect young children in the households of the participants.
- Patients will be instructed to discuss taking any medication, including over the counter medication and herbal preparations, with the research staff and study doctor prior to taking the medication in order to reduce risks.
- In terms of the risks of discontinuing previous medications, subjects will be advised of the risks of this prior to giving informed consent. In addition, patients will monitored weekly at clinic visits during washout.
- To preserve patient safety, patients will be withdrawn from the study if they receive CGI Improvement scores of 6 ("much worse") or 7 ("very much worse") for two consecutive visits. If a patient receives a CGI-I score of 6 or 7, they will be re-evaluated within 1 week, and if the score remains at 6 or 7, the patient will be discontinued from the study.
- In addition, patients will be withdrawn if they develop significant suicidal ideation (as measured by HDRS item 3 scores of 3 or 4) or psychotic symptoms (as measured by HDRS item 19 (3 or greater), HDRS item 20 (2 or greater), and psychiatrist judgment.
- Patients who receive consistent CGI-Improvement scores of 4 ("no change) or higher (indicating worsening) at each visit up to and including week 6 will complete the Acute Phase at week 6 and will be entered into the continuation phase at that point.
- In general, study medication will be decreased by 30 mg/d every 4 days, with each dose level being maintained for 4 days before lowering.

MRI study

- Patients will be told they can request to stop the MRI scan if they experience any discomfort.
- Patients are screened for metal objects prior to scanning as well as medicinal patches that may be conductive and cause discomfort. If metal objects are present or if a patch cannot be removed, the scan will not be performed.
- Female patients of childbearing potential will have a urine pregnancy test performed within 7 days prior to each scan, and the scan will not be performed if the test is positive.

6. Describe benefits to subjects, if any.

Patients can receive information about their health from any physical examinations and laboratory tests to be done in this study. Participation in this study might benefit subjects by helping to reduce symptoms and improve psychosocial functioning. Patients will not receive copies of their MRI scans but will be told of any important medical findings that are discovered.

7. Confidentiality: Describe means by which privacy will be protected and confidentiality of data maintained. Include procedures for the storage and

protection of electronic data. NOTE: If a Certificate of Confidentiality will be obtained for this study, please indicate.

All data with identifying information will be stored in locked cabinets or files. Data to be analyzed will have identifying information removed and subject codes will be used. The identity of patients will not be revealed in presentations or publications of the data from this study. All data that is stored or transmitted electronically will have identifying information removed. MRI data from this study will be stored on computers behind firewalls in Dr. Bradley Peterson's laboratory at NYSPI. All members of the study team, including research assistants, will be educated about the importance of adhering to strict patient confidentiality procedures.

V. Attestations:

[A] I agree to the following:

1. **I have carefully reviewed this proposal for completeness and for compliance with local and federal regulatory requirements related to the protection of human research subjects.**
2. **All named co-investigators have agreed to their involvement in the protocol as proposed.**
3. **Any financial interests that study investigators and those documenting consent have in relation to the study sponsor and/or any products under study have been disclosed and forwarded to the EAB for review under separate cover.**
4. **All study staff with a significant role in the design or implementation of the human subject components of this protocol have completed CITI training in human research subject protections. Associated documentation is in my files.**
5. **All members of the research team are appropriately qualified to carry out their roles.**
6. **I will notify the IRB of any serious and/or unexpected adverse events and any other events that occur during the course of study participation that have or might have significant impact on the rights, welfare, or safety of study participants.**
7. **No changes will be made to the protocol without the prior written approval of the NYSPI-IRB. Any deviations from the approved protocol or consent procedure will be reported promptly to the IRB.**

____________________________	__________________________	________
**Principal Investigator (Print Name)**	**Signature**	**Date**
____________________________	__________________________	________
**Faculty Sponsor (if necessary) (Print Name)**	**Signature**	**Date**

[B] I agree to the following:

1. **The proposal has been prepared and reviewed by appropriately qualified members of the department's staff, and the investigators are appropriately qualified to carry out the study.**
2. **I approve the use of departmental space and resources to carry out this study.**
3. **I have reviewed and approved this protocol for submission to the IRB.**

______________________________	____________________________	________
**Research Division Chief (Print Name)**	**Signature**	**Date**

[C] For studies involving PET procedures: I have reviewed and approved this protocol for submission to the IRB.

_____________________________	_______________________	________
**Responsible Investigator (Print Name)**	**Signature**	**Date**

Table 4. Schedule of events in protocol.

SCHEDULE OF EVENTS	Acute Phase (Double Blind)								Continuation Phase (Open-Label)					
	V1	V2	V3	V4	V5	V6	V7	V8 or Early D/C	V9	V10	V11	V12	V13	V14 Or early d/c
Visit Week	Screen	0	1	2	4	6	8	10	11	12	14	16	18	22
SCID, entry criteria	X													
Psychiatric and medical history, physical exam, **EKG**[f]	X													
Lab tests	X	X[a]						X[C]						X[c]
MRI scan (incl. metal screening, and urine pregnancy test for women)[e]	X							X						X[d]
Patient Scales A: BDI, SCL, PCGI, MOTCS, ABS, ASEX, BPI		X			X		X	X			X		X	X
Patient scales B: TCI, SAS, CBAS, EWPS		X						X						X
HDRS, CGI	X	X	X	X	X	X	X	X	X	X	X	X	X	X
CDRS, GAFS		X			X		X	X			X		X	X
Dispense study drug		X	X	X	X	X	X	X	X	X	X	X	X	
Break blind[b]								X						
AE and concom meds		X	X	X	X	X	X	X	X	X	X	X	X	X
Check Compliance			X	X	X	X	X	X	X	X	X	X	X	X
Vitals	X	X	X	X	X	X	X	X	X	X	X	X	X	X

[a] optional, perform if a retest is needed

[b] the patient will be assigned a new doctor who will confidentially break the blind

[C] only cytokines and a.m. cortisol will be collected

[d] For subjects who have met criteria for responder while taking duloxetine

[e] for eligible subjects only

[f] EKG for subjects >=60 years only
